# UROPA: a tool for Universal RObust Peak Annotation

**DOI:** 10.1038/s41598-017-02464-y

**Published:** 2017-06-01

**Authors:** Maria Kondili, Annika Fust, Jens Preussner, Carsten Kuenne, Thomas Braun, Mario Looso

**Affiliations:** 1 Max Planck Institute for Heart and Lung Research, Bioinformatics Core Unit (BCU), Ludwigstrasse 43, 61231 Bad Nauheim, Germany; 2 Max Planck Institute for Heart and Lung Research, Department of Cardiac Development and Remodeling, Ludwigstrasse 43, 61231 Bad Nauheim, Germany

## Abstract

The annotation of genomic ranges of interest represents a recurring task for bioinformatics analyses. These ranges can originate from various sources, including peaks called for transcription factor binding sites (TFBS) or histone modification ChIP-seq experiments, chromatin structure and accessibility experiments (such as ATAC-seq), but also from other types of predictions that result in genomic ranges. While peak annotation primarily driven by ChiP-seq was extensively explored, many approaches remain simplistic (“most closely located TSS”), rely on fixed pre-built references, or require complex scripting tasks on behalf of the user. An adaptable, fast, and universal tool, capable to annotate genomic ranges in the respective biological context is critically missing. UROPA (**U**niversal **RO**bust **P**eak **A**nnotator) is a command line based tool, intended for universal genomic range annotation. Based on a configuration file, different target features can be prioritized with multiple integrated queries. These can be sensitive for feature type, distance, strand specificity, feature attributes (e.g. protein_coding) or anchor position relative to the feature. UROPA can incorporate reference annotation files (GTF) from different sources (Gencode, Ensembl, RefSeq), as well as custom reference annotation files. Statistics and plots transparently summarize the annotation process. UROPA is implemented in Python and R.

## Introduction

Many bioinformatic analyses result in the definition of genomic regions of interest, generated by a variety of methods. Minimally, they consist of a chromosome, a start position, and an end position, but can also contain a range of additional data such as the strand of the region. For this type of data, the Browser Extensible Data (BED, https://genome.ucsc.edu/FAQ/FAQformat#format1) format became the *de facto* standard format, and a wide range of tools that are able to handle and produce this datatype were developed. In the context of the extensively used chromatin immunoprecipitation and sequencing (ChIP-seq) method, the regions of interest are commonly referred to as peaks, denoting regions of high coverage of reads produced by the experiment. Peaks are generated by computational tools named “peak callers” (such as MACS2^1^ or MUSIC^2^) and denote potential binding sites of the protein under investigation. Frequently, investigated proteins are transcription factors (TF) or histones with specific modifications. In order to interpret these binding sites, a set of peak annotation tools such as Homer^3^, Goldmine^4^, GREAT^5^ or ChIPpeakAnno^6^ were developed. As these tools are mainly focused on the assignment of TFs or histone modifications to the corresponding gene, they apply methods to calculate the closest distance of a peak to the transcription start site of genes located in the direct neighborhood.

**Figure 1:**
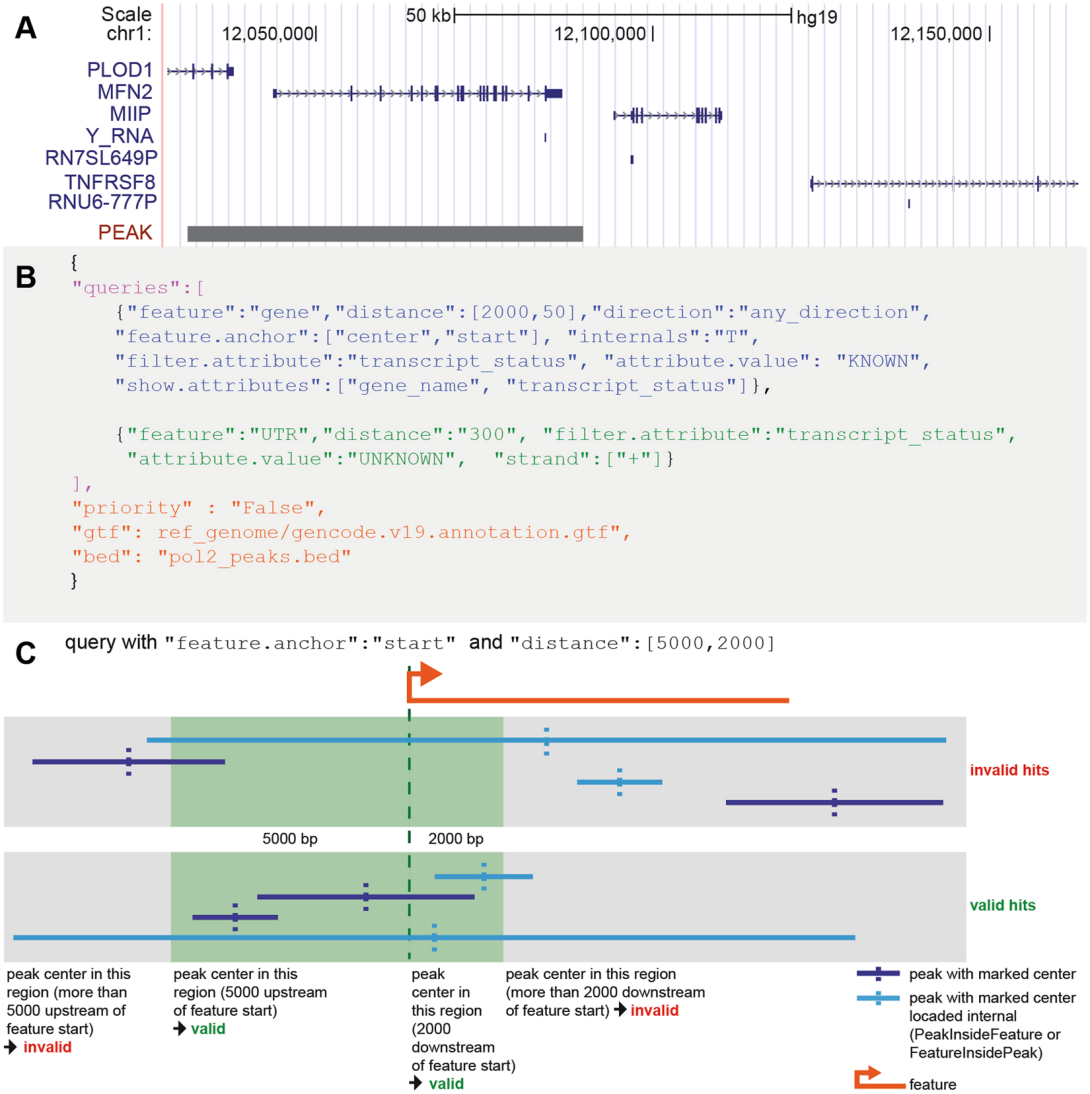
(**A**) Example of a complex annotation situation: region of interest (peak, black bar) overlaps multiple candidate features (blue). These include protein coding genes (PLOD1, MFN2, MIIP, TNFRSF8) with exon (block) and intron (line) structure and non-coding genes (Y-RNA, RN7SL649P, RNU6-777P). Depending on the origin of the peak region the optimal annotation will vary. (**B**) Example on JSON formatted configuration file with two queries: I) begin and end of query section (purple); II) first query targeting gene features with multiple conditions and output filters (key:value pairs, blue); III) second query relating to UTR features (green); IV) global parameters on input files and prioritization. (**C**) Query and feature scheme: Illustration of an oriented feature (orange) and peaks (light and dark blue) that are filtered according to a query with asymmetrical distances as given on top. According to this query, green indicates the valid region around the queried start anchor of the feature. Dark blue peaks centered outside of the green region are never assigned to the feature (upper row, “invalid hits”). Dark blue as well as light blue peaks centered in the green region are assigned to the feature (lower row, “valid hits”). If “internals” key of the query is set to TRUE, light blue peaks given in the upper row are assigned to the feature.

However, annotation of peaks can become complex (Fig. 1A), especially in regions where multiple genes are located in close proximity or if features are supposed to be treated preferentially based on their category or relative localization.

The biological origin of peaks can render the annotation step even more complex. In an epigenetics context, the ChIP-seq method is often used to interpret the state of chromatin by the parallel investigation of a number of different histone modifications such as methylation, acetylation or phosphorylation on distinct sites^7^. These marks of open or condensed chromatin are known to influence transcription regulation. In addition, the combination of such histone marks can be used to identify regulatory regions like enhancers, which can be located up to 1 Mb distant from the gene they regulate, thus requiring special care considering optimal annotation.

While ChIP-seq peaks are one-dimensional in relation to the protein of interest, more recently developed methods are intended to produce information about the global structure of the chromatin (ATAC-seq, FAIRE-seq, DNase-seq, Mnase-seq), giving a detailed footprint on regions that are accessible or closed. However, peaks resulting from such methods implicitly include additional information such as TF and histone binding patterns^8^. In order to meaningfully interpret these data, the annotation of these peaks needs to be adapted to combine e.g. genes, TF binding sites and other known regulatory regions.

Here, we present UROPA (**U**niversal **RO**bust **P**eak **A**nnotator) as a versatile tool to annotate peaks or other genomic regions. UROPA supports a JavaScript Object Notation (JSON) based configuration file format (Fig. 1B) for simple application, optionally incorporating multiple annotation queries. The tool permits linkage of individual queries including prioritization. Arbitrary features in the reference annotation file can be addressed in a granular way, such as absolute searches for overlaps or searches with distance thresholds for start, center or end position of individual features. Filtering on additional annotation columns in the reference annotation file is supported as well. UROPA generates publication ready graphical statistics on peak annotation rates, feature distribution, and query assessment (Additional File 1).

## Results

### Overview

**Figure 2:**
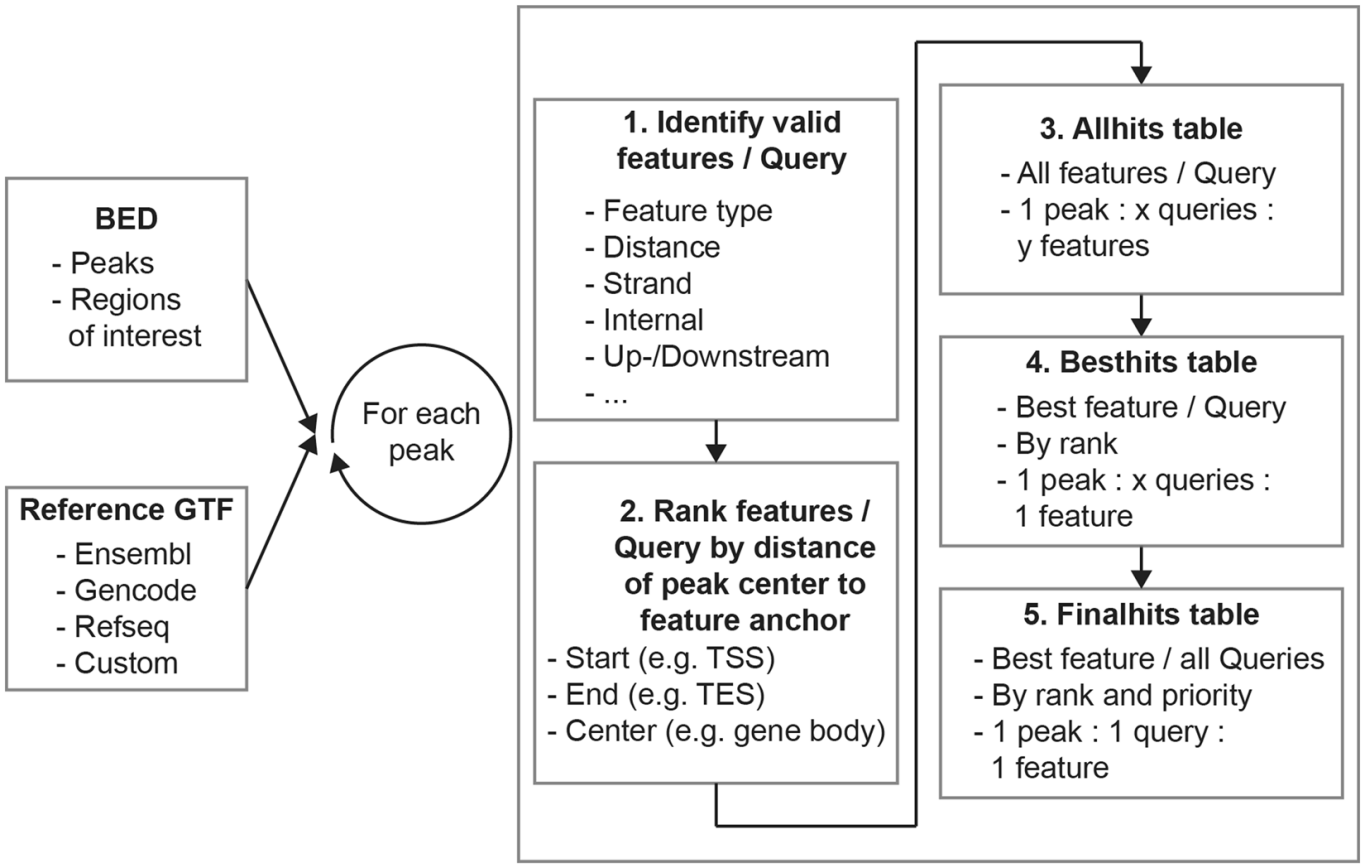
Outline of the UROPA algorithm. (1) For each peak all queries are consecutively checked for features satisfying various optional criteria. (2) The resulting candidate features are ranked for each query based on the distance of the peak center to the feature anchor(s) of interest (e.g. start, end, center of the feature). (3) All candidate features resulting from any query are stored in the”all hits” table. (4) The best candidate feature for each query is stored in the “best hits” table. (5) Only the one best feature among all queries is stored in the “final hits” table. This step can optionally include prioritization of queries to ensure a desired precedence (e.g. prefer protein_coding genes even if they are located farther away from the peak). These three output files cover various granularities considering the desired outcome.

UROPA is a freely available command line application depending on three files: a configuration file (JSON format), a reference annotation file in General Feature Format (GTF, http://www.ensembl.org/info/website/upload/gff.html) and a file containing genomic ranges (BED format, e.g. peaks). Beside the widely used genomic annotation files from Gencode, Ensembl, or RefSeq, any custom reference information can be included here, as long as it adheres to the GTF specification (a script to generate GTF files from tab delimited files is provided: see UROPA_to_GTF section in methods). In order to permit simple configuration even for complex annotation problems (Fig. 1C), we chose JSON format for the configuration file as shown in (Fig. 1B). Individual queries can be generated that are interpreted one by one for each individual peak under investigation based on the algorithm outlined in Fig. 2.

### Multi query support and prioritization

The queries listed in the configuration file defined by the user are flexible statements that allow filtering for all values found in a reference annotation file. UROPA can parse custom generated GTF files with arbitrary keywords as well (see annotation examples below). Within each query line, the user defines filters as *key*:*value* pairs. These independent pairs are linked with a logical AND operation within each row. If multiple values are present for a specific key, these values can be inquired by a list operator (Fig. 1B). Valid keys are the *features* (e.g. exon, gene, etc.) and *attributes* (e.g. gene_type, gene_status, etc.) of interest, the *distance* that should be used for scanning, the *feature anchor* that should be taken into account for distance calculation, and the *strand* of a peak. The *feature anchor* key specifies the region of a feature that should be used for annotation (e.g. start, end, or center of the feature). This option represents a valuable improvement considering peaks which are located at the end of a gene (*feature anchor*:*end*; e.g. histone modification H3K9me3^9^), or throughout gene bodies (*feature anchor:center*; e.g. H3K36me3^9^). The default annotation mode for many present tools focuses only on the distance to the transcription start site (TSS) (*feature anchor:start*).

By default, the set of queries specified by the user is connected by an OR operator, attributing the same weight to all queries and giving precedence to the most closely located feature of any query. This behavior can be changed by the global *priority* flag, leading to a hierarchical processing of queries. Here, the first query resulting in a valid candidate feature for a peak will abort any further searches for this peak. This annotation mode is highly versatile, as it permits annotation of peaks with respect to quality levels (e.g. increasing distance sets with respect to TSS), preferred categories (e.g. first protein_coding, then lncRNA, etc.), or exclusive assignments (either genic or intergenic). In order to achieve high quality annotations, two identical queries could be defined with respect to the TSS, that differ considering *distance*. The first one would be defined with a very strict/small distance, the latter with a more relaxed value. In combination with *priority*:true, two groups of peaks would be generated in the result files, one reflecting peaks that are located directly on the TSS (query 0), the second group located in moderate proximity (no hit for query 0, only a hit for query 1). The exclusive priority feature is unique to UROPA (see Table 1).

**Table 1:** Comparison matrix of popular annotation tools: the first column defines features supported by the respective tools given in column 2–5. Available/not available features are coded as Y or N, respectively. In case of comparable features, explanations/details are given as key words. *Indicates “only via additional programming”.

	Homer	GREAT	ChiPpeakAnno	Goldminer	UROPA
Annotation Database	Refseq	UCSC (internal database)	Pre-calculated sets, e.g. “EnsDb.Hsapiens.v75”	All genomic range files	All GTF formatted files
Helper script to generate annotation file	assignGenomeAnnotation	N	N	makeGRanges()	UROPAtoGTF-tool
Target for distance calculation	TSS only	TSS only	Start/Center/End of selected feature	Overlap	Start/Center/End of selected feature
Select feature type in annotation file	N	N	Y	(N)*	Y
Limitation on characteristics, e.g. “protein_coding”	N	N	N	(N)*	Y
Definition of multiple annotation queries	N	N	N	N	Y
Prioritization of queries	N	N	(Y) no exclusive ranking (precedence)	(Y) within gene model context, but not globally	Y
Limit results to upstream/downstream of selected features	N	N	N	N	Y
Granularity of resulting annotations	N	N	Shows all hits, no aggregation to the best hit	Clear result structure, but single best hit is often missing	All hits, best hits per query, and merged best hits among all queries
Parallelization	N	N	N	N	Y
Simple customizing (no programming)	N	Y (only in web-based version)	N	N	Y
Audience	Bioinformatician/Biologist	Biologist	Bioinformatician	Bioinformatician	Bioinformatician/Biologist
Definition of distance cutoff	N	N	Y	N	Y

### Relative localization

UROPA can handle a range of cases considering the relative localization of peaks and features versus each other. The strand of peaks can be 1) ignored to retrieve target features on both strands, 2) honored to only retrieve features on the same strand, or 3) can be interpreted to only retrieve features on the opposite strand. These options establish compatibility with various experimental protocols.

Additionally, we introduced a *direction* key to be set to *upstream* or *downstream*, enabling the assignment of ranges depending on their relative location before or behind a target feature. This option can assist to assign cis-regulatory elements such as transcription factor binding sites to nearby TSS, enforcing candidate genes to be located downstream of the binding site.

Another useful parameter called *internals* optionally ignores the distance calculation versus candidate features if the peak center is located inside the feature (or vice versa). This exception is supposed to handle cases where large genes fail the distance filter, despite completely overlapping a peak. This option can result in reported distances greater than the maximum distance permitted. The result files report on internal location of the peak inside a feature and vice versa (see also Fig. 1C).

### Granularity and further result customization

The interacting attributes *filter attribute* and *attribute value* are unique to UROPA and allow another filtering step during the annotation. Those two keys concern the attributes column of the reference annotation file. The *filter attribute* corresponds to different attributes within this column, e.g. gene_biotype, and the *attribute value* equates to the value of this attribute, e.g. protein coding. Together these can be used to limit the features returned to those of a certain category.

The two main result files of the tool provide summarizing steps that can either include multiple valid candidate features for the selected queries intended for more complex filtering tasks that the user may apply later, or a final annotation of only one feature per peak (Additional File 1, Table 1 to 4). These tables can optionally be condensed further to summarize all valid candidate annotations per peak in one row. If multiple queries are given in the configuration file, an additional intermediate result file is generated, providing the best feature annotation for each query and for each respective peak.

**Figure 3:**
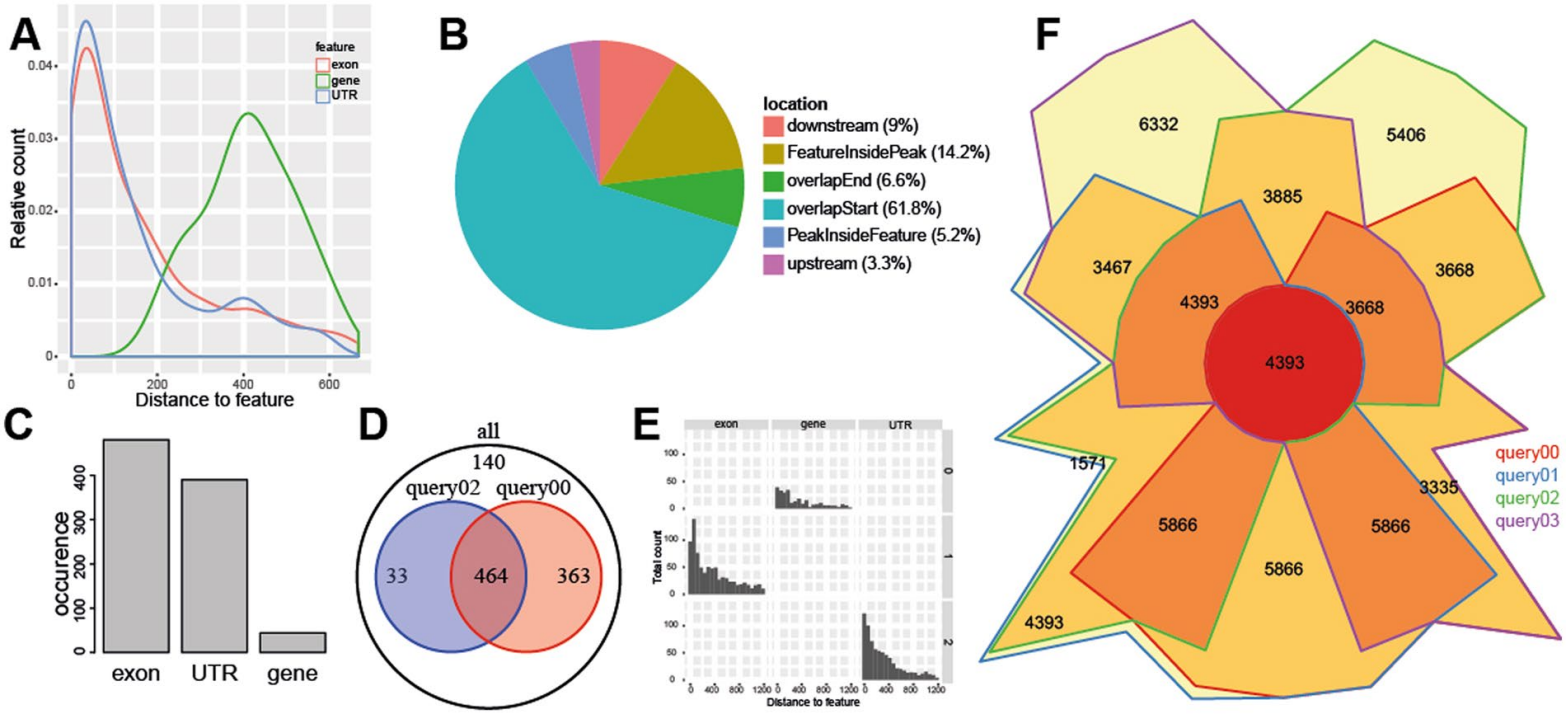
UROPA graphical summary report. (**A**) The distance to the feature anchor is displayed as a fraction of the total peaks annotated using a density plot. This information can be useful to determine optimal distance settings for annotation. (**B**) Relative localization of peaks in relation to the annotated feature (one pie chart plot per feature). (**C**) Bar plot of total occurrence of individual features (one plot per query). (**D**) All queries are included in a pairwise comparison to show possible overlaps. Assuming multiple concurrent queries, the amount of exclusively and commonly annotated peaks can be deduced. (**E**) Distance histogram in relation to query and feature where each query is depicted separately. (**F**) The Chow Ruskey plot represents an area-proportional Venn diagram. It reveals the distribution of peaks that could be annotated per query and works for up to 5 queries.

The output annotation columns can be selected by the user. As indicated in Fig. 1B, the show.attributes key allows the definition of columns that should be reported in the result files. Furthermore, UROPA optionally generates a global summary report, visualizing characteristics of annotations for a set of input peaks. It generates a number of charts summarizing the occurrences for each feature, the distance for each feature, and the relative position of peaks (Fig. 3A–C). If UROPA was configured with multiple queries, additional plots comparing the characteristics and overlaps of the individual queries are generated (Fig. 3D–F). Together, these graphics represent a valuable instrument to interpret the annotation, and can serve to define optimal parameters for future analyses.

While built-in filter functions of UROPA focus on the distance of the peak center to a target feature, the software furthermore computes overlap percentages between peak and feature, and vice versa. These values can be employed for downstream analyses by the user to perform additional filtering on the annotation candidates to enforce selected amounts of intersection. This can be useful in the ChIP-seq context, e.g. if peaks of a TF are annotated with a custom reference annotation file hosting the location of TF binding sites as features, where a complete overlap of the pattern and peak marks high quality hits.

### Comparison and benchmarking

In order to demonstrate the universal character as well as the novel features of UROPA, we selected the well-established tools Homer, GREAT, Goldmine and ChIPpeakAnno for a detailed feature comparison and benchmarking (Table 1). As illustrated, UROPA not only combines a set of individual features given by one or some of the other tools in one place, but also considerably extends the functionality of peak annotation processes by features like “free selection of feature characteristics”, “definition of multiple additive or concurrent queries”, “exclusive prioritization”, “limitation to up/downstream locations” and “computational parallelization”. While some of these functions can be manually scripted with methods of existing tools, UROPA reduces the effort by the user significantly, as it is designed to be used without programmatic access and fully encapsulates complex workflows.

As UROPA is intended to be able to annotate arbitrary regions of interest in a universal manner, it can be configured to simulate the behavior of other annotation tools. We performed a pairwise comparison to the tools named above with a published peak file resulting from a POLR2A ChIP-seq experiment (14989 peaks, see Methods) to prove that we can cover a wide range of existing functionality.

**Figure 4:**
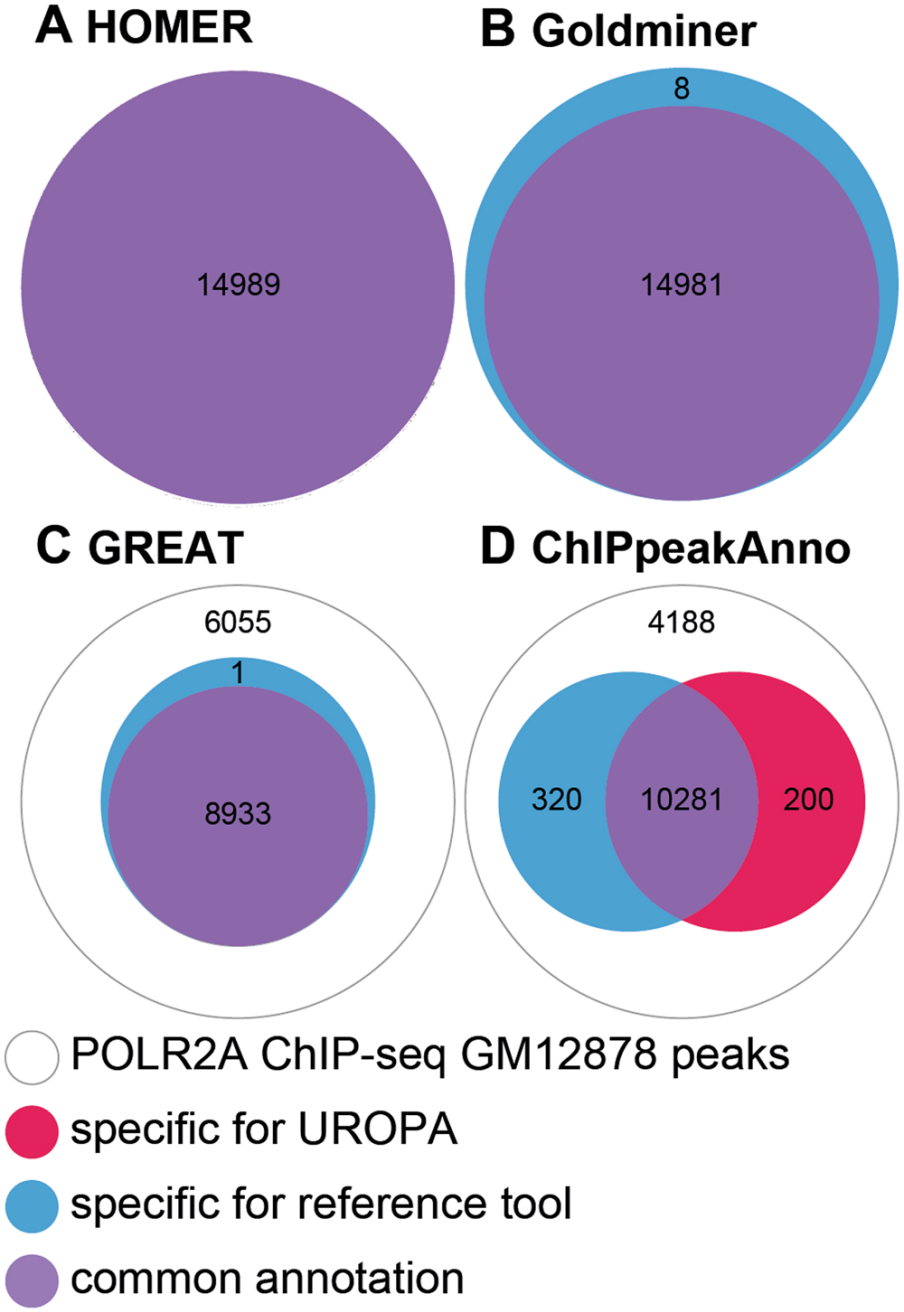
Global comparison of UROPA to other peak annotation tools. White circles represent peaks without any annotation, blue circles represent the number of peaks exclusively annotated by the respective tool, red circles represent peaks exclusively annotated by UROPA, and violet circles represent peaks annotated by both tools. (**A**) Comparison of UROPA and Homer, no tool specific peaks are reported. (**B**) Comparison of UROPA and Goldminer. (**C**) Comparison of UROPA and GREAT. (**D**) Comparison of UROPA and ChIPpeakAnno.

In our first comparison we used the full reference annotation file of Homer and generated a custom reference file from it to perform annotation with UROPA. As Homer annotates all peaks via the closest feature without any distance limits, we configured UROPA accordingly. As shown in Fig. 4A, all peaks were annotated via both tools. When we checked for the respective hits for each peak, we found a perfect annotation overlap for 100% of the peaks. Due to these results, we conclude that UROPA is able to simulate the behavior of Homer.

In our second comparison to Goldmine, we used the Gencode hg19 version as reference annotation file. It was downloaded directly from the Gencode website for UROPA, while the getUCSCTable() function was used for Goldmine. We used one query targeting the feature gene, activated the *internals* parameter and set a distance threshold of 100000 for UROPA in order to simulate the basic operation mode of Goldmine (“target closest gene TSS; count overlapping gene as a hit ignoring distance”). As indicated in Fig. 4B, the results of the tools are very similar, as only a small number of 8 peaks were exclusively annotated by Goldmine. We found all these peaks to be located in a greater distance than demanded by our threshold. When we had a closer look on the single peak annotations, we found seven peaks assigned to different target genes (see Additional File 2). For six of the genes assigned to these peaks we found no entry in the Gencode version from the website, indicating slight differences between the annotation files. One peak (peak 6230) was annotated to be intergenic with a distance >100000 bp to the next gene in Goldmine, although it overlaps the start position of TLN2, which was assigned via UROPA. Furthermore, we found a considerable number of peaks (~10600) annotated with a distance of 0 in the Goldmine result file that were reported with larger distances in the UROPA result file (see Additional File 2). From these, we found ~6% to be >10000 bp away, containing two extreme examples (peak_11600 and peak_13322, Additional File 2) with a distance of 260766 and 116902 to the TSS by UROPA respectively.

For comparison to GREAT, we utilized the reference annotation file provided at the GREAT homepage for UROPA. Again, we designed the query of UROPA in order to simulate the basal plus extension mode of operation of GREAT (see methods). As indicated in Fig. 4C, UROPA showed an identical annotation for 99% of the peaks, while ~40% were not annotated by both tools. All annotated peaks were assigned to the identical genes. Due to these results, we conclude that UROPA can simulate the algorithm of GREAT.

Finally, we benchmarked UROPA against ChIPpeakAnno, a tool that is capable to utilize arbitrary reference annotation files and supports extensive variability considering available parameters. We used an Ensembl based reference annotation file (Homo_sapiens.GRCh37.75), and a comprehensive configuration file (see Methods). As shown in Fig. 4D, the tools annotated ~72% of all peaks. Within the annotated peaks, we found a large overlap of ~95%, while peaks exclusively annotated by UROPA and ChIPpeakAnno accounted for 2–3% of the total. When we investigated the exclusive peak groups, we found all 200 peaks exclusive to UROPA to be located downstream of features. We thus assume that ChIPpeakAnno removes peaks located downstream of features, ignoring the selected distances. Most of the 320 peaks exclusive to ChIPpeakAnno were located inside or overlapping a feature. As ChIPpeakAnno reports a distance of 0 for these peaks, we were not able to further evaluate this group. However, when we performed a second comparison (Additional File 3) with our *internals:true* key, we were able to reduce the number of ChIPpeakAnno specific peaks to 103, while the UROPA specific peak number increased to 3166 peaks. In this comparison the latter number was comprised of ~95% of peaks that are located inside of features and ~5% were the already identified downstream assignments. Within the group of annotated peaks, all peaks refer to identical features.

While we used the same peak file for all comparisons, we found the total number of annotated peaks to be variable (Fig. 4C and D outer circles). This is assumed to result from differences considering reference annotation files and annotation strategies. Although we were not able to generate a 100% overlap with all reference peak annotation softwares, the benchmark demonstrates that UROPA subsumes basic functions of popular tools.

### Complex use case

**Figure 5:**
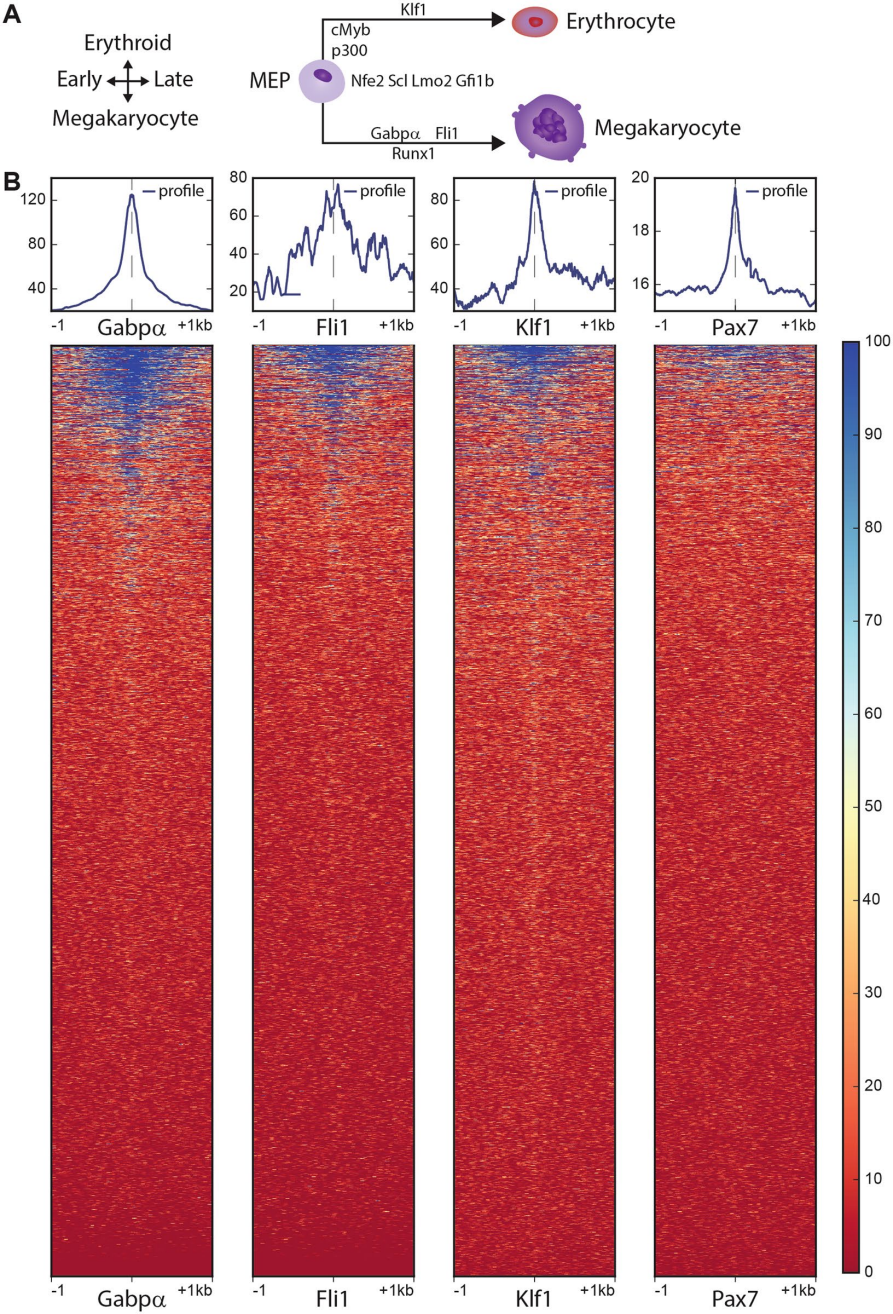
(**A**) Megakaryocyte differentiation, from left to right: Megakaryocyte progenitor cells (MPC) differentiate to erythrocytes (upper branch) or megakaryocytes (lower branch), while specific transcription factors for each branch drive the differentiation process (figure adapted from^10^). (**B**) Heatmaps on four in silico predicted transcription factor binding sites in the mm10 mouse genome assembly. Binding sites are located in the center, surrounded by the ATAC read signal from −1 kb to +1 kb. A globally normalized color scale represents the strength of the respective ATAC signal. The binding profile is shown at the top of each heatmap. Heatmaps from the left to the right represent Gabp-alpha, Fli1and Klf1 as Megakaryocyte progenitor specific transcription factors and Pax7 as Megakaryocyte unrelated factor.

Recent method development in the epigenetics context gave rise to applications that permit the investigation of chromatin structure and accessibility. Assay for Transposase Accessible Chromatin with high-throughput sequencing (ATAC-seq) is one of the latest developments able to report on open chromatin regions. We chose a public dataset from ENCODE (see methods) on megakaryocyte erythroid progenitor (MEP) cells. The progenitor cells give either rise to mature megakaryocytes or erythroid cells. The first lineage specification is driven by a transcription factor (TF) cocktail comprising Runx1, Gabp-alpha and Fli1, while the latter is driven by c-Myc, p300 and Klf1^10^ (Fig. 5A).

The origin of this ATAC-seq dataset suggests that regions with open chromatin will include binding sites of megakaryocyte related TFs and that these binding sites should be located in proximity to protein coding genes. In order to test this assumption, we predicted all potential binding sites for selected TFs (Gabp-alpha, Fli1, Klf1) in the mouse genome *in silico* and visualized the degree of accessible chromatin around these potential binding positions with heat maps and profile plots (Fig. 5B). As illustrated, megakaryocyte related TFs revealed a clear set of binding sites with accessible chromatin (upper part of the heatmaps, colored dark blue) as well as binding sites that are completely closed (colored in red). Furthermore, the heatmap shows a differing width of the opened chromatin locations, varying from almost 2 kb (upper heatmap) to smaller ranges of ~200 bp (width of dark blue signal). As a control, we included transcription factor Pax7, which is not intended to play a role in megakaryocyte development but is indicated in fetal development and cancer growth^11^. As expected, the predicted potential Pax7 binding sites were mostly found to be closed (Fig. 5B right heatmap). The general degree of chromatin accessibility at the respective sites (shown in the ATAC-seq coverage pattern above heatmaps, Fig. 5B) is at least a 4 fold higher for the MEP related TFs when compared to the Pax7 control. In order to test our hypothesis expecting proximity of open binding sites to protein coding genes, we clustered the individual heatmaps and generated lists of open and closed potential TF binding sites, respectively. As functional open TF binding sites should be mostly located within the promotor, we annotated the respective lists of genomic positions with an advanced UROPA configuration. We made use of multiple promotor definitions concerning size and localization (small symmetric regions around the TSS and larger asymmetric regions upstream of the TSS (see Methods) in order to unravel the location of TF binding sites in an unbiased way. As expected, we found a large percentage (up to 92%) of the open TF binding sites in the classical promotor regions of protein coding genes, while the corresponding closed TF binding sites are depleted for promotor regions (≪11%). Table 2 exemplarily illustrates the distribution of promotor associated binding sites for Gabp-alpha in the open chromatin (c1) and closed chromatin (c2) regions, while the remaining TFs are listed in Additional File 4. Correspondingly, we examined the results on the Pax7 control TF. We clustered for open and closed binding sites again and annotated the respective groups via UROPA as described before. Not surprisingly, we found a significantly smaller number of open sites that could be assigned to promotors (30–45% for c1, Additional File 4), indicating a more randomized set of open chromatin locations compared to the megakaryocyte related TFs. In the closed group c2 of Pax7, a similarly low percentage of sites were assigned to promotors as in case of the megakaryocyte related TFs.

**Table 2:** Gabp-alpha associated, in silico predicted binding sites: Binding sites are clustered by ATAC-seq signal into open (c1) and closed (c2) regions. For each query indicated as rows, the percentage of peaks with respective annotation is listed. Query 0 to 4 reflect typical promotor definitions, query 5 reflects open intergenic regions. Table is based on the best per query result file.

Query	Feature - filter attribute	Anchor	Distance	Gabpa	
				Cluster 1	Cluster 2
**0**	gene - protein coding	start	1000:500	86	4
**1**	gene - protein coding	start	2000:500	87	6
**2**	gene - protein coding	start	3000:500	88	8
**3**	gene - protein coding	start	5000:500	89	11
**4**	gene - pseudogene	start	5000:500	1	2
**5**	gene - protein coding	start	100000	100	78

### Performance

As annotation is a reoccurring analysis step applied for many use cases, UROPA supports parallel processing in order to balance computational load and runtime. As shown for a large BED file with >100000 peaks and a complex query set (Additional File 5), multithreading efficiency approximates a linear trend. For our performance test, we utilized one to sixteen cores on a single socket machine while RAM assignments were constant for all runs. As it was not possible to adjust all tools to perform an identical comparison task, excluding hard-coded additional computations, we decided to not include misleading runtime benchmarks. Nonetheless, we noted that UROPA using 16 threads was approximately twice as fast as the fastest competitor (Goldmine) when annotating peaks with the closest TSS based on Gencode genes, excluding one-time indexing/caching procedures.

## Discussion

The correlation of enrichment peaks resulting from ChIP-seq/ATAC-seq or other sequencing based techniques with known features for annotation purposes is a central task for bioinformatics pipelines in the field. Many downstream analyses steps rely on the successful attribution of peaks to the relevant genetic features, which serve as connectors to facilitate pathway enrichment analyses, transcription correlation, network analyses, and other secondary analyses. Especially peaks deriving from histone ChIP-seq experiments present a challenge here, as histone modifications can result in data with variable profiles, targeting not only TSS of genes, but also gene bodies or transcription termination site. Furthermore, different reference annotation files exist which can vary considering gene definitions^12^. Depending on the biological background under investigation, the best reference genes are not necessarily the total set, as non-coding genes may be of lesser interest when compared to protein coding genes or vice versa. Additionally, many peaks do not allow a clear attribution to exactly one reference feature, as multiple valid candidate features may be in range.

The most popular tools for this class of tasks are arguably Homer and GREAT, which are limited to annotations focused on TSS of the most closely located gene and use fixed reference annotation files that can only be changed with considerable effort. The annotation of peaks resulting from histone modifications not targeting the TSS will invariably lead to inaccurate assignments using these tools. When comparing an annotation run relative to the TSS with another one focusing on the gene center for histone modification H3K36me3, which is mostly enriched in the gene body, we found that ca. 25% of the peaks were annotated with a different gene when using the full Gencode annotation for reference (Analysis of GEO dataset GSM733733, Additional File 6). ChIPpeakAnno and Goldmine can be adjusted to hone such constraints, but require a certain level of proficiency in the R statistical programming language. Finally a range of other atomic tools like BEDTOOLS^13^ and BEDOPS^14^ could be chained to effect similar results as provided by UROPA. However, utilizing these tools requires extensive scripting, to combine outputs of multiple separate runs.

Apart from classical peaks resulting from sequencing based methods as stated above, other genomic ranges of interest exist, where classical peak annotation tools fail. One common example in the epigenetics context are CpG sites, known to be targeted by methyltransferases in order to activate or repress gene transcription. Using UROPA, CpG sites can be easily classified hierarchically as promotor, gene internal, or distal based on three queries.(i: promotor region upstream of protein coding genes; ii: gene body of protein coding genes itself with distance to feature center is zero and internals key set to TRUE; iii: all genes without distance limitation upstream and downstream) and priority key set to TRUE. As a result, one would receive a final annotation file, where each CpG site is classified hierarchically as promotor (i), gene internal (ii), or distal (iii). Similar annotation or classification steps might be carried out for single nucleotide permutations (SNPs). UROPA can be applied with even greater flexibility when employing custom generated reference annotation files. The reference annotation file could host e.g. TFBS for multiple TFs to annotate open chromatin regions derived from ATAC-seq. Another possible application is the correlation of peaks of multiple histone modifications with each other to identify and classify enhancers.

While there can be a certain amount of complexity involved in setting up a configuration file for a specific task in UROPA, there will be considerable time savings when compared to scripting the underlying agglomerations manually using existing tools. Extensive tutorials are included with UROPA bearing a number of examples for reasonable configurations. As UROPA is fully implemented in Python (only the additional visualization features use R), the installation process is straightforward, while computational load and runtime can easily be adjusted to match the computational resources available.

Summarizing, UROPA can replace popular tools for peak annotation while considerably extending the functionality and reducing the effort by the user (see Table 1). Particularly ChIP-seq and ATAC-seq datasets can profit from a more dynamic annotation incorporating the specific properties of the respective experiment.

## Methods

### UROPA tool

The tool UROPA was implemented in Python and R. Additionally, we integrate the open-source package tabix^15^ for indexing genomic ranges. R is mainly employed for the summary statistics, utilizing the libraries ggplot2^16^, gplots^17^, gridExtra^18^, jsonlite^19^, VennDiagram^20^, and Vennerable^21^ in their latest version. If multiprocessing is used, the package snow^22^ is needed.

The pipeline is freely available for local execution from our online source repository located at https://github.molgen.mpg.de/loosolab/UROPA. Any GTF formatted reference annotation file as well as any BED formatted regions of interest file can be used as input data. A detailed explanation of all input and output files is available at our online documentation http://uropa.readthedocs.io/en/latest/. The UROPA project is licensed under the MIT license.

### Benchmarking

For tool comparison and benchmarking, we utilized a POLR2A peak file (ENCFF001VFA, assembly hg19, UCSC, version 1.2 from https://www.encodeproject.org/).

#### Homer

The annotatePeaks.pl program from Homer Version 4.7 was used for this comparison. The full annotation file provided during installing Homer (data/genomes/hg19/hg19. full.annotation) was reformatted to GTF format (uropa.to.gtf.R) and used for the UROPA annotation run. Since Homer does not support distance settings, UROPA was also started with the default distance, the query is given below:

$$ \mbox{``} queries\mbox{''}:[\{ \mbox{``} show.attributes\mbox{''}: \mbox{``} homer.anno\mbox{''}, \mbox{``} feature.anchor\mbox{''}: \mbox{``} start\mbox{''}\}]$$

#### GREAT

The GREAT run was performed via the web interface (http://bejerano.stanford.edu/great/public/html/) in ‘basal plus extension’ mode with the following parameters: Proximal 5.0 kb upstream, 5.0 kb downstream, plus Distal up to 0.0 k (upstream and downstream of TSS). As the resulting annotation file contained annotations with a distance larger than 5 kb, we manually removed these to achieve equivalence with UROPA. For the UROPA run, we selected the available gene annotation used by GREAT (available at http://bejerano.stanford.edu/help/download/attachments/2752609/hg19.great3.0.genes.txt? version=1&modificationDate=1443465966000&api=v2) after reformatting (uropa.to.gtf.R). The configuration file for UROPA is given below:

$$\begin{array}{c} \mbox{``} queries\mbox{''}:[\{ \mbox{``} feature\mbox{''}: \mbox{``} gene\mbox{''}, \mbox{``} distance\mbox{''}:{5000}, \mbox{``} show.attributes\mbox{''}\\ : \mbox{``} gene\_name\mbox{''}, \mbox{``} feature.anchor\mbox{''}: \mbox{``} start\mbox{''}\}]\end{array}$$

#### ChIPpeakAnno

ChIPpeakAnno supports all reference annotation files available in R, e.g. the package Homo_sapiens.GRCh37.75. This file needed translation to GRanges prior usage. The function *annoPeaks (peaks, annoGR, bindingType* = “*startSite*”, *bindingRegion* = *c*(−*5000*, *5000*), *ignore.peak.strand* = *TRUE*) was used for the comparison to UROPA. Homo_sapiens.GRCh37.75 is identical to the Ensembl GTF file, which was used for the UROPA annotation with the following query:

$$\begin{array}{c} \mbox{``} queries\mbox{''}:[\{ \mbox{``} feature\mbox{''}: \mbox{``} gene\mbox{''}, \mbox{``} distance\mbox{''}:{5000}, \mbox{``} show.attributes\mbox{''}\\ :[ \mbox{``} gene\_name\mbox{''}, \mbox{``} gene\_id\mbox{''}], \mbox{``} feature.anchor\mbox{''}: \mbox{``} start\mbox{''}\}]\end{array}$$

For the comparison displayed in the Additional File 3, the *internals* key was set to TRUE.

### UROPA to GTF

The preprocessing tool was implemented in R to transform files that do not adhere to the standard GTF file format. Files intended for reformatting need to include a header line with information about the genomic location (chromosome, start, and end), and may provide further standard GTF columns. Given columns are kept while missing columns are filled with dots. All columns not required for the GTF format will be combined and kept as the attribute column. The tool can either transform one file or a folder of files. Running the tool requires the input file(s) and accepts three more parameters (source, feature, and threads).

Example command line call:

$$\begin{array}{c}Rscript\,UROPAtoGTF.R\,directory/of/various/files/source\\ \quad =UCSC\,feature=TFBS\,threads={5}.\end{array}$$

### Use case ATAC-seq

ATAC-seq FASTQ/BAM/BIGWIG/BED files were downloaded from www.encodeproject.org/ (accession ENCSR229QKB). The replicates were aligned using STAR^23^.

Position weight matrices obtained from JASPAR^24^ were utilized for TFBS prediction in the mouse genome (assembly mm10) *in silico*. Briefly, all positions across the mouse genome with a 95% match for respective TFs were identified utilizing the R packages TFBSTools^25^ and JASPAR 2016^26^. TF binding patterns related to megakaryocyte progenitor cells (Gabp-alpha(MA0062.2), Fli1 (MA0475.2), Klf1 (MA0493.1)) and megakaryocyte unspecific TF Pax7 (adapted from^27^ were used. Predicted binding sites were analyzed by extracting the corresponding ATAC-seq coverage with deepTools^28^. To distinguish between open and closed chromatin from these regions including a TF binding pattern, kmeans clustering on the ATAC-seq coverage values was applied. Open and closed sites were extracted and annotated with promotors utilizing UROPA. The analysis was performed for all TFs in parallel. The configuration for the annotation with UROPA was defined as followed:

$$\begin{array}{c} \mbox{``} queries\mbox{''}:[\{ \mbox{``} feature\mbox{''}: \mbox{``} gene\mbox{''}, \mbox{``} distance\mbox{''}:[{1000},{500}],\\  \mbox{``} feature.anchor\mbox{''}: \mbox{``} start\mbox{''},filter.attribute\mbox{''}: \mbox{``} gene\_biotype\mbox{''},\\  \mbox{``} attribute.value\mbox{''}: \mbox{``} protein\_coding\mbox{''}, \mbox{``} show.attributes\mbox{''}\\ :[ \mbox{``} gene\_name\mbox{''}, \mbox{``} gene\_id\mbox{''}]\},\end{array}$$

$$\begin{array}{c}\{ \mbox{``} feature\mbox{''}: \mbox{``} gene\mbox{''}, \mbox{``} distance\mbox{''}:[{2000},{500}], \mbox{``} feature.anchor\mbox{''}: \mbox{``} start\mbox{''},\\  \mbox{``} filter.attribute\mbox{''}: \mbox{``} gene\_biotype\mbox{''}, \mbox{``} attribute.value\mbox{''}: \mbox{``} protein\_coding\mbox{''}\},\end{array}$$

$$\begin{array}{c}\{ \mbox{``} feature\mbox{''}: \mbox{``} gene\mbox{''}, \mbox{``} distance\mbox{''}:[{3000},{500}], \mbox{``} feature.anchor\mbox{''}: \mbox{``} start\mbox{''},\\  \mbox{``} filter.attribute\mbox{''}: \mbox{``} gene\_biotype\mbox{''}, \mbox{``} attribute.value\mbox{''}: \mbox{``} protein\_coding\mbox{''}\},\end{array}$$

$$\begin{array}{c}\{ \mbox{``} feature\mbox{''}: \mbox{``} gene\mbox{''}, \mbox{``} distance\mbox{''}:[{5000},{500}], \mbox{``} feature.anchor\mbox{''}: \mbox{``} start\mbox{''},\\  \mbox{``} filter.attribute\mbox{''}: \mbox{``} gene\_biotype\mbox{''}, \mbox{``} attribute.value\mbox{''}: \mbox{``} protein\_coding\mbox{''}\},\end{array}$$

$$\begin{array}{c}\{ \mbox{``} feature\mbox{''}: \mbox{``} gene\mbox{''}, \mbox{``} distance\mbox{''}:[{5000},{500}], \mbox{``} feature.anchor\mbox{''}: \mbox{``} start\mbox{''},\\  \mbox{``} filter.attribute\mbox{''}: \mbox{``} gene\_biotype\mbox{''}, \mbox{``} attribute.value\mbox{''}: \mbox{``} pseudogene\mbox{''}\},\end{array}$$

$$\begin{array}{c}\{ \mbox{``} feature\mbox{''}: \mbox{``} gene\mbox{''}, \mbox{``} filter.attribute\mbox{''}: \mbox{``} gene\_biotype\mbox{''},\\  \mbox{``} attribute.value\mbox{''}: \mbox{``} protein\_coding\mbox{''}\}]\end{array}$$

Evaluation of binding site localization was based on the best per query result file.

### Availability of data and material

All data used for testing and comparison as well as example data for the UROPA algorithm and UROPA itself can be downloaded from the public github repository: https://github.molgen.mpg.de/loosolab/UROPA.

## Electronic supplementary material

Supplemental Info File
