# Supplementary material for: UROPA: a tool for Universal RObust Peak Annotation
Source: Sci Rep. 2017 Jun 1;7:2593. doi: 10.1038/s41598-017-02464-y (PMC5453960; doi:10.1038/s41598-017-02464-y)
Supplement: Supplementary file 1 — Supplemental Info File [file 41598_2017_2464_MOESM1_ESM.pdf]

Additional Informations

UROPA: a tool for **U**niversal **RO**bust **P**eak **A**nnotation

Maria Kondili [1#] maria.kondili@mpi-bn.mpg.de

Annika Fust [1#] annika.fust@mpi-bn.mpg.de

Jens Preussner [1] jens.preussner@mpi-bn.mpg.de

Carsten Kuenne [1] carsten.kuenne@mpi-bn.mpg.de

Thomas Braun [2*] thomas.braun@mpi-bn.mpg.de

Mario Looso [1*] mario.looso@mpi-bn.mpg.de

[1]Max Planck Institute for Heart and Lung Research, Bioinformatics Core Unit (BCU), Ludwigstrasse 43, 61231 Bad Nauheim, Germany

[2]Max Planck Institute for Heart and Lung Research, Department of Cardiac Development and Remodeling, Ludwigstrasse 43, 61231 Bad Nauheim, Germany

*Corresponding author

#Contributed equally

Additional File 1: Exemplary summary statistics and peak annotation result files generated by UROPA; The file represents the summary statistics as reported by UROPA for the configuration file shown on page one, followed by the detailed summary visualizations. Details can be found in the manual: http://uropa-anual.readthedocs.io/output.html#summary-visualisation

{ "queries":[
{"feature":"gene", "distance":5000, "feature.anchor":"start", "filter.attribute":"gene_type", "attribute.value":"protein_coding", "internals":"True", "show.attributes":["gene_name","gene_type"]},
{"feature":"gene", "distance":10000, "feature.anchor":"start", "filter.attribute":"gene_type", "attribute.value":"protein_coding", "direction":"upstream"},
{"feature":"transcript", "distance":1000, "feature.anchor":"start", "internals":"False"}],
"priority":"False",
"gtf": "gencode.v19.annotation.gtf",
"bed": "ENCFF001VFA.peaks.bed" }

peak_id	peak_chr	peak_start	peak_center	peak_end	feature	feat_start	feat_end	feat_strand	feat_anchor	distance	genomic_location	feat_ovl_peak	peak_ovl_feat	gene_name	gene_type	query
peak_1	chr21	26932550	26945254	26957959	NA	NA	NA	NA	NA	NA	NA	NA	NA	NA	NA	0
peak_1	chr21	26932550	26945254	26957959	NA	NA	NA	NA	NA	NA	NA	NA	NA	NA	NA	1
peak_1	chr21	26932550	26945254	26957959	NA	NA	NA	NA	NA	NA	NA	NA	NA	NA	NA	2
peak_16	chr1	149856066	149858333	149860600	gene	149856010	149858232	-	start	101	overlapStart	0.48	0.98	HIST2H2BE	protein_coding	0
peak_16	chr1	149856066	149858333	149860600	gene	149859019	149859466	-	start	1133	FeatureInsidePeak	0.1	1	HIST2H2AB	protein_coding	0
peak_16	chr1	149856066	149858333	149860600	gene	149858525	149858961	+	start	192	FeatureInsidePeak	0.1	1	HIST2H2AC	protein_coding	0
peak_16	chr1	149856066	149858333	149860600	gene	149859440	149872351	+	start	1107	overlapStart	0.26	0.09	BOLA1	protein_coding	0
peak_16	chr1	149856066	149858333	149860600	gene	149859440	149872351	+	start	1107	overlapStart	0.26	0.09	BOLA1	protein_coding	1
peak_16	chr1	149856066	149858333	149860600	transcript	149858525	149858961	+	start	192	FeatureInsidePeak	0.1	1	HIST2H2AC	protein_coding	2
peak_16	chr1	149856066	149858333	149860600	transcript	149856010	149858232	-	start	101	overlapStart	0.48	0.98	HIST2H2BE	protein_coding	2

Additional Table 1: Allhits output of UROPA annotation with config file as described above. Multiple hits for each query for each peak are included (see column query).

peak_id	peak_chr	peak_start	peak_center	peak_end	feature	feat_start	feat_end	feat_strand	feat_anchor	distance	genomic_location	feat_ovl_peak	peak_ovl_feat	gene_name	gene_type	query
peak_1	chr21	26932550	26945254	26957959	NA	NA	NA	NA	NA	NA	NA	NA	NA	NA	NA	0,1,2
peak_16	chr1	149856066	149858333	149860600	gene	149856010	149858232	-	start	101	overlapStart	0.48	0.98	HIST2H2BE	protein_coding	0

Additional Table 2: Finalhits output of UROPA annotation with config file as described above. Only best hit for each peak is given (see column query).

peak_id	peak_chr	peak_start	peak_center	peak_end	feature	feat_start	feat_end	feat_strand	feat_anchor	distance	genomic_location	feat_ovl_peak	peak_ovl_feat	gene_name	gene_type	query
peak_1	chr21	26932550	26945254	26957959	NA	NA	NA	NA	NA	NA	NA	NA	NA	NA	NA	0
peak_1	chr21	26932550	26945254	26957959	NA	NA	NA	NA	NA	NA	NA	NA	NA	NA	NA	1
peak_1	chr21	26932550	26945254	26957959	NA	NA	NA	NA	NA	NA	NA	NA	NA	NA	NA	2
peak_16	chr1	149856066	149858333	149860600	gene	149856010	149858232	-	start	101	overlapStart	0.48	0.98	HIST2H2BE	protein_coding	0
peak_16	chr1	149856066	149858333	149860600	gene	149859440	149872351	+	start	1107	overlapStart	0.26	0.09	BOLA1	protein_coding	1
peak_16	chr1	149856066	149858333	149860600	transcript	149856010	149858232	-	start	101	overlapStart	0.48	0.98	HIST2H2BE	protein_coding	2

Additional Table 3: Besthits output of UROPA annotation with config file as described above. Best hit for each query for each peak is given (see column query).

peak_id	peak_chr	peak_start	peak_center	peak_end	feature	feat_start	feat_end	feat_strand	feat_anchor	distance	genomic_location	feat_ovl_peak	peak_ovl_feat	gene_name	gene_type	query
peak_1	chr21	26932550	26945254	26957959	NA,NA,NA	NA,NA,NA	NA,NA,NA	NA,NA,NA	NA,NA,NA	NA,NA,NA	NA,NA,NA	NA,NA,NA	NA,NA,NA	NA,NA,NA	NA,NA,NA	0,1,2
peak_16	chr1	149856066	149858333	149860600	gene,gene,transcript	149856010, 149859440, 149856010	149858232, 149872351, 149858232	-,+,-	start,start,start	101, 1107, 101	overlapStart,overlapStart,overlapStart	0.48,0.26,0.48	0.98,0.09,0.98	HIST2H2BE,BOLA1,HIST2H2BE	protein_coding,protein_coding,protein_coding	0,1,2

Additional Table 4: Besthits_compact output of UROPA annotation with config file as described above. This file will be generated if multiple queries are defined and the "-r" parameter is added in the command line call. Best hits per query are combined to one row per peak.

UROPA summary

Input:

query	feature	distance	feature.anchor	internals	strand	direction	filter.attribute	attribute.value	show.attributes
query00	gene	5000	start	True	ignore	any_direction	gene_type	protein_coding	c("gene_name", "gene_type")
query01	gene	5000	start	False	ignore	upstream	gene_type	protein_coding	None
query02	transcript	1000	start	False	ignore	any_direction	None	None	None

config_key	specification
priority:	False
bed:	ENCFF001VFA.peaks.bed
gtf:	gencode.v19.annotation.gtf

Results:

query	peaks	peaks_with_annotation
query00	14989	4050
query02	14989	7198

UROPA annotated 11248 peaks.
Not all queries represent final hits!

1. Distances of annotated peaks in finalhits:

The following density plot displays the distance of anntotated peaks to its feature(s) based on the finalhits.

Additional Info:
This is independent of the number of queries,
all features present in the finalhits are displayed.

Note on output files:

allhits: All candidate features resulting from any query
(1 peak : x queries : y annotations)

besthits: All candidate features resulting from any query
(1 peak : x queries : 1 annotation)

finalhits: Only the one best feature among all queries
(1 peak : 1 query : 1 annotation)

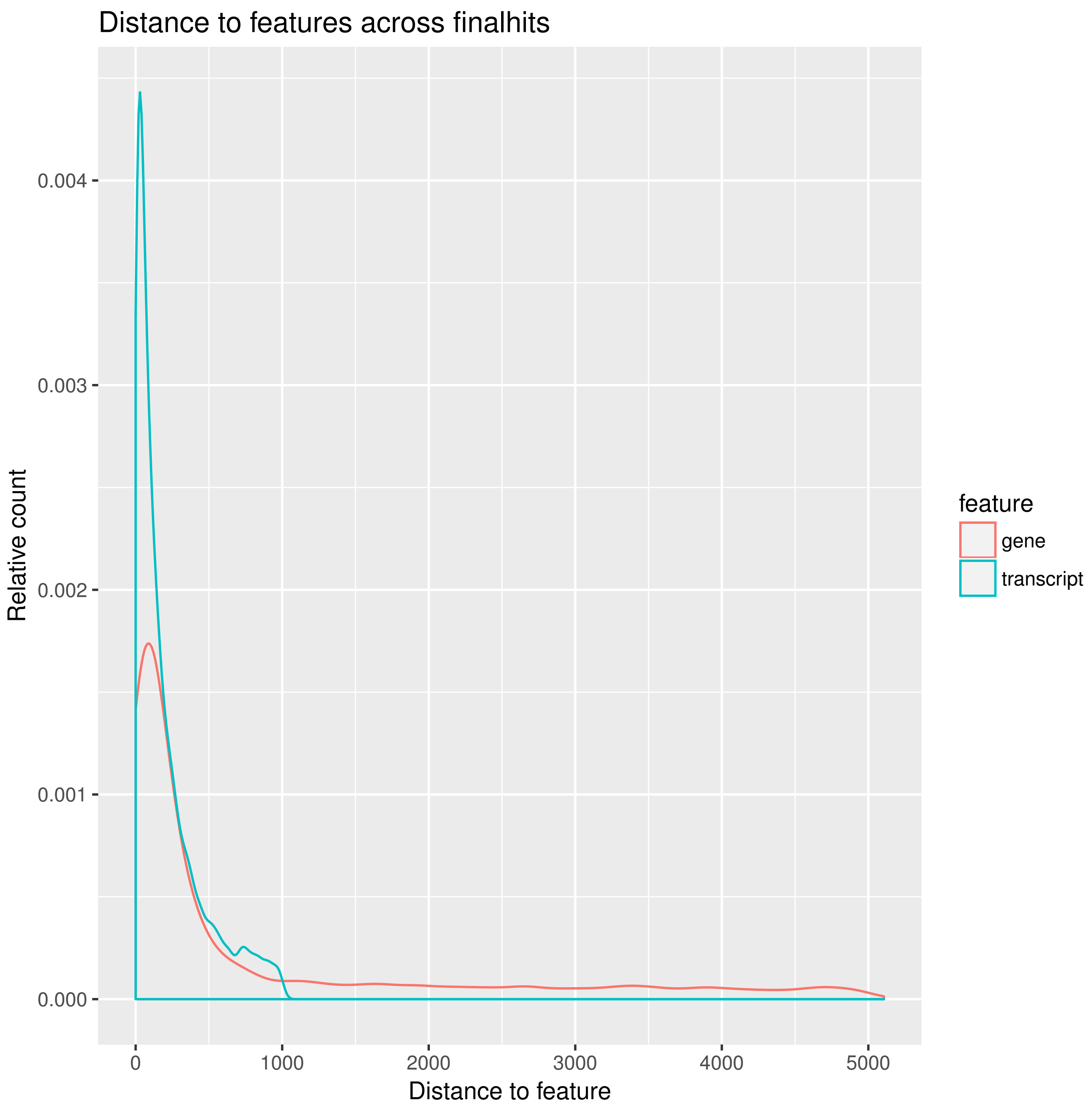

Distance to features across finalhits
Relative count
0.000
0.001
0.002
0.003
0.004
0
1000
2000
3000
4000
5000
Distance to feature
feature
gene
transcript

2. Relative locations of annotated peaks to features in finalhits:

The following pie chart(s) illustrate the relativ location of the peaks
in relation the respective annotated feature as represented in the finalhits
The best feature found amoung all of the queries is used for this plot.

Additional Info:
This is independent of the number of queries,
all represented features of the finalhits are displayed.

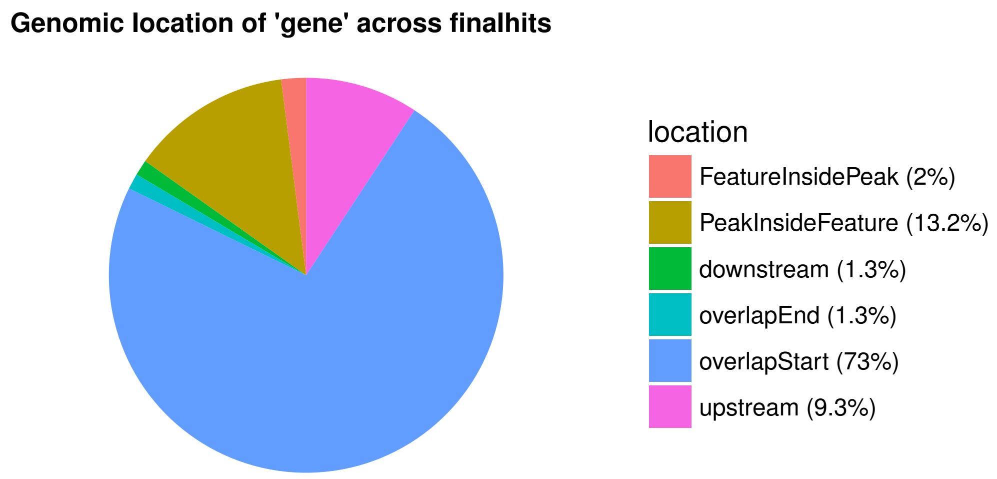
Genomic location of 'gene' across finalhits
location
FeatureInsidePeak (2%)
PeakInsideFeature (13.2%)
downstream (1.3%)
overlapEnd (1.3%)
overlapStart (73%)
upstream (9.3%)

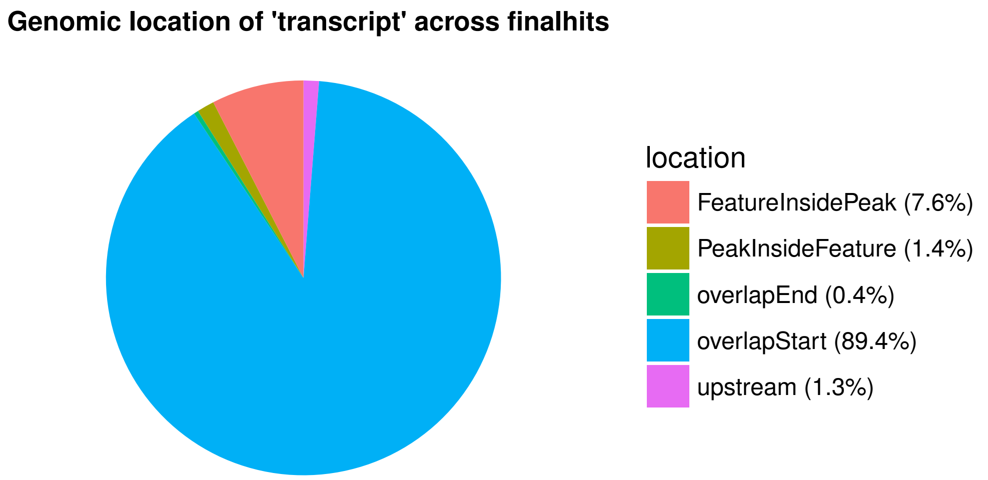
Genomic location of 'transcript' across finalhits
location
FeatureInsidePeak (7.6%)
PeakInsideFeature (1.4%)
overlapEnd (0.4%)
overlapStart (89.4%)
upstream (1.3%)

3. Allocation of available features in finalhits:

Bar plot displaying the occurrence of the different features if there is more than one feature assigned for peak annotation based on the finalhits. The best annotation found amoung all of the queries is used for this plot.

Additional Info:
This is independent of the number of queries;
all features present in the finalhits are displayed.

If only one feature is present, this plot will be skipped.

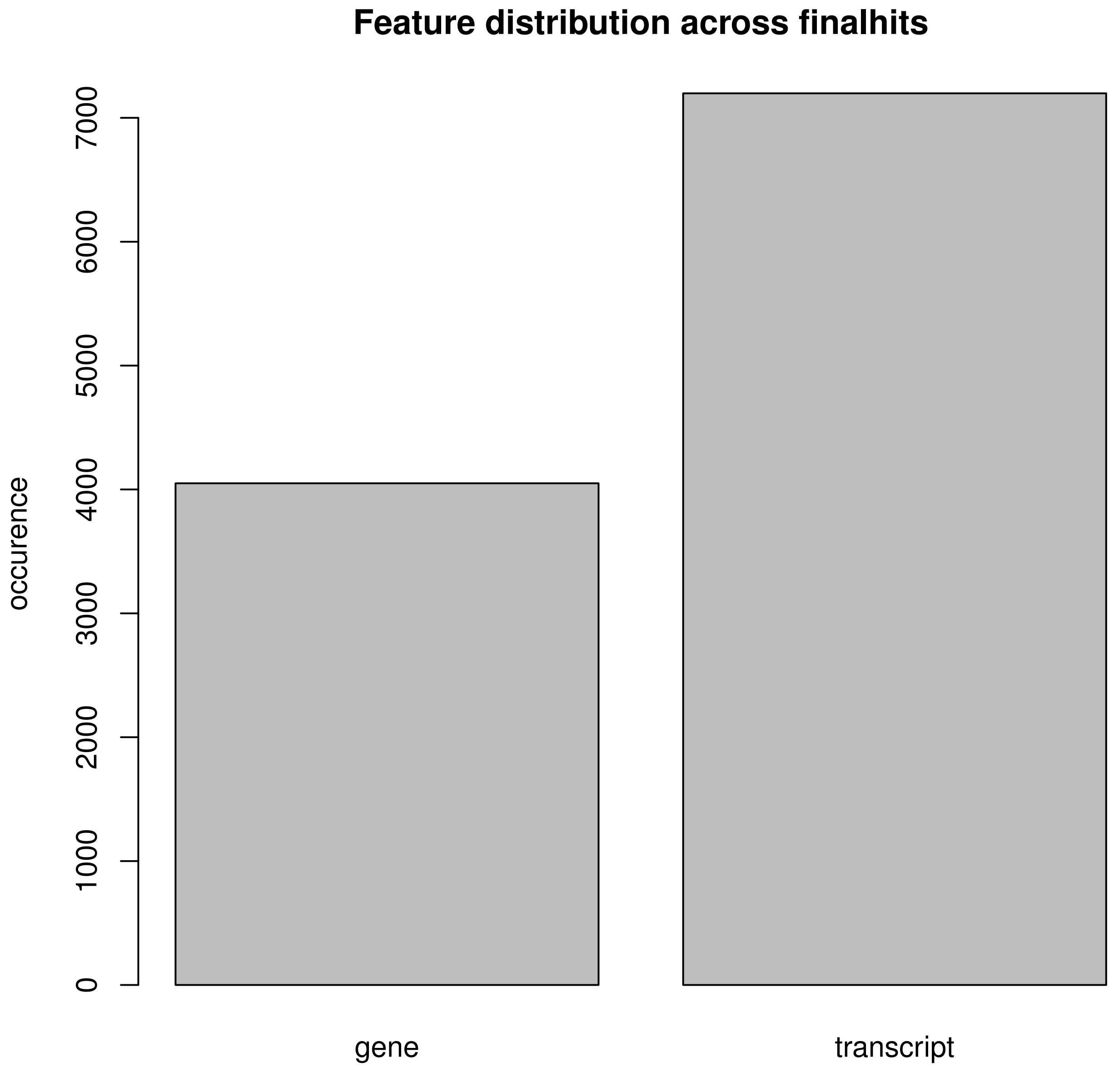

Feature distribution across finalhits
occurence
0
1000
2000
3000
4000
5000
6000
7000
gene
transcript

4. Distances of annotated peaks seperated for features and queries in besthits:

The distribution of the distances per feature per query
is displayed in histograms based on the besthits.

Additional Info:
This is dependent on the number of queries;
all features present in any query are displayed.

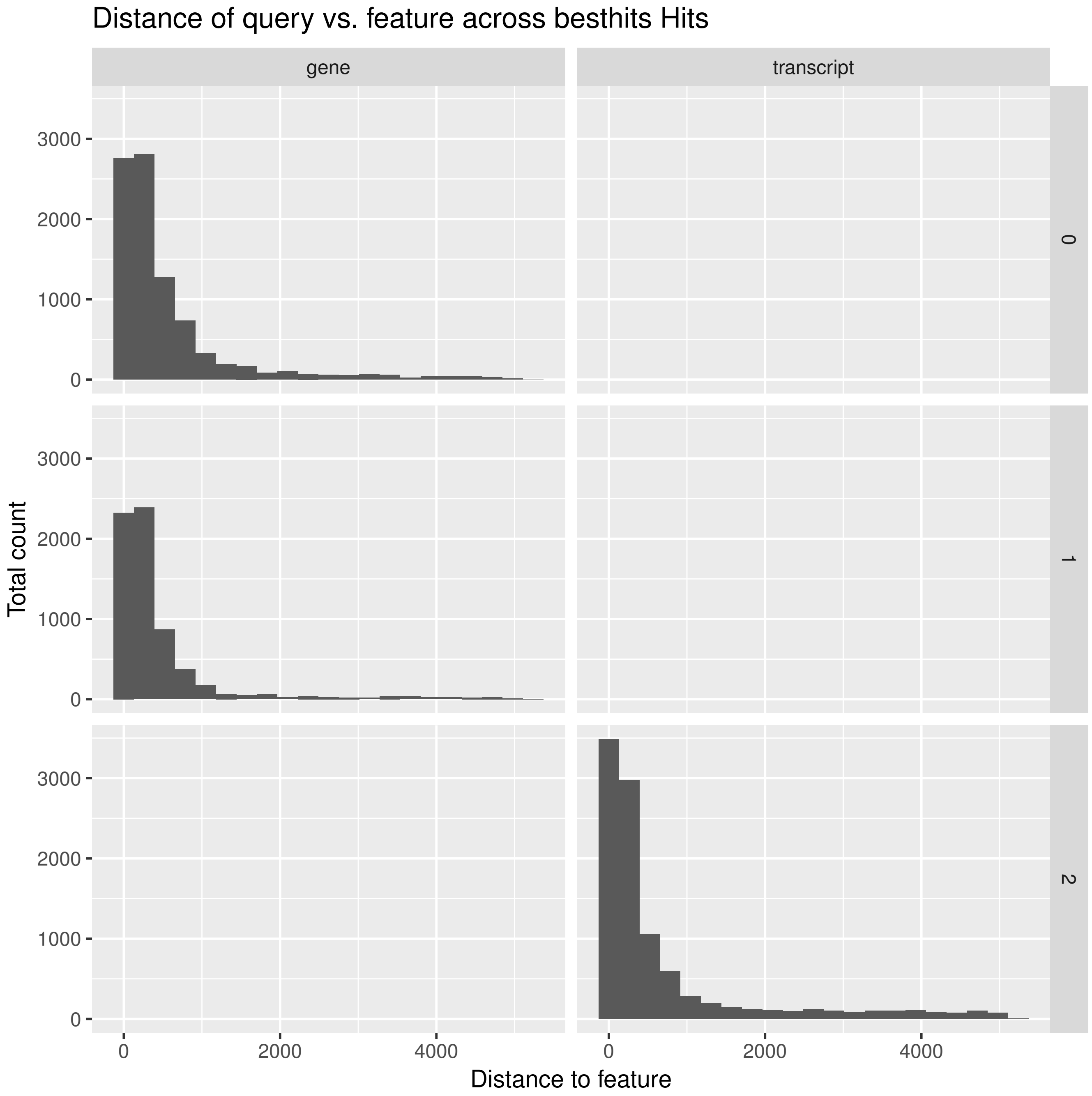

Distance of query vs. feature across besthits Hits
gene
transcript
0
1
2
Total count
3000
2000
1000
0
0
2000
4000
Distance to feature

5. Relative locations of annotated peaks in besthits:

The following pie chart(s) illustrate the relativ location of
the peaks in relation the respective annotated feature as represented in the besthits.

Additional Info:
This is dependent on the number of queries;
all features present in the besthits are displayed.

Genomic location of 'gene' across besthits Hits

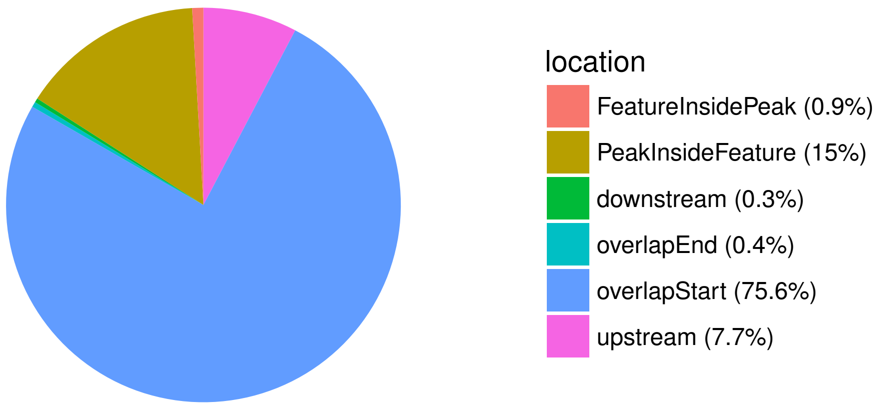

Genomic location of 'transcript' across besthits Hits

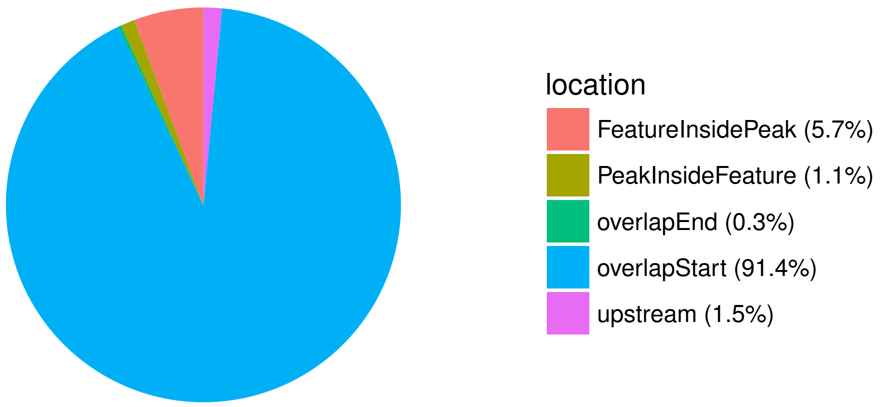

6. Allocation of available featurs in besthits:

Bar plot displaying the occurrence of the different features if there is more than one feature assigned for peak annotation based on the besthits. The best annotation found in each query is used for this plot

Additional Info:
This is independent of the number of queries,
all features present in the besthits are displayed.

If only one feature is present,
this plot will be skipped.

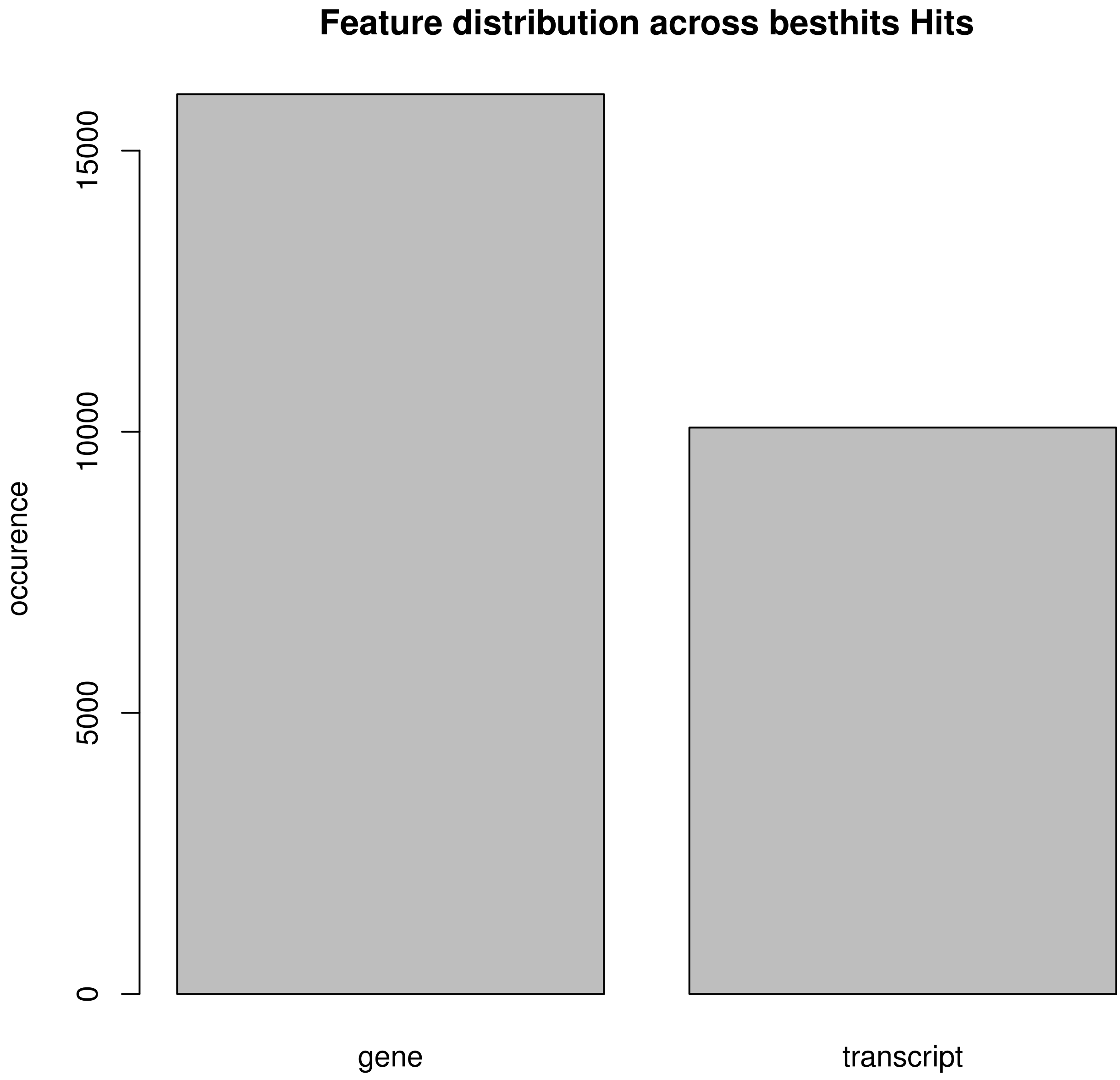
Feature distribution across besthits Hits
occurence
0
5000
10000
15000
gene
transcript

7: Pairwise comparisons of query annotations:

The following venn diagrams display peak based pairwise comparisons among all queries based on the besthits.

Additional Info:
If only one query is present,
this plot will be skipped.

Peak based pairwise compare of query00 and query02

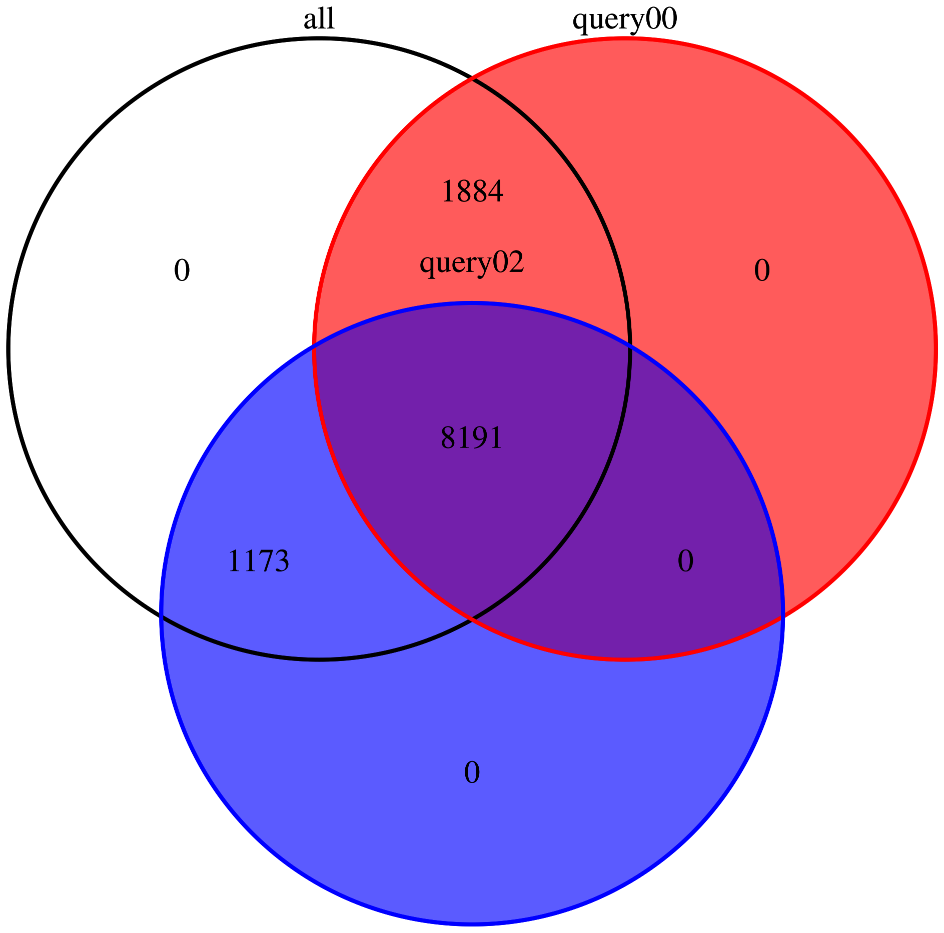

Peak based pairwise compare of query00 and query01

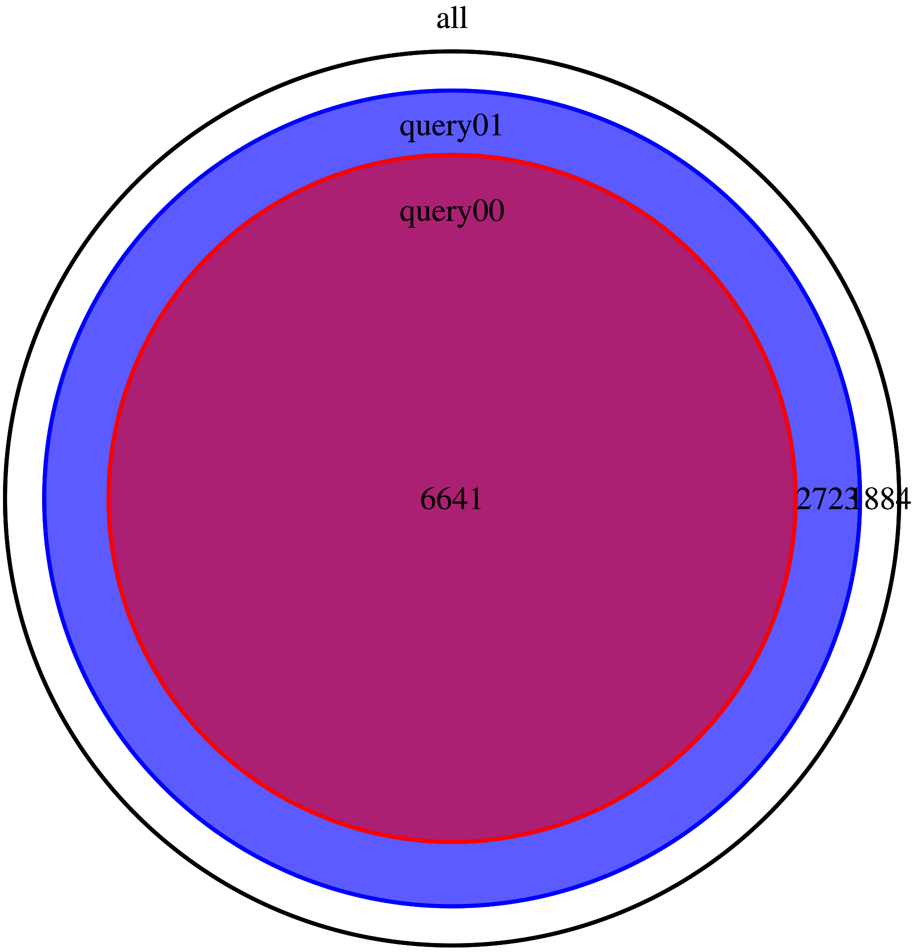

Peak based pairwise compare of query01 and query02

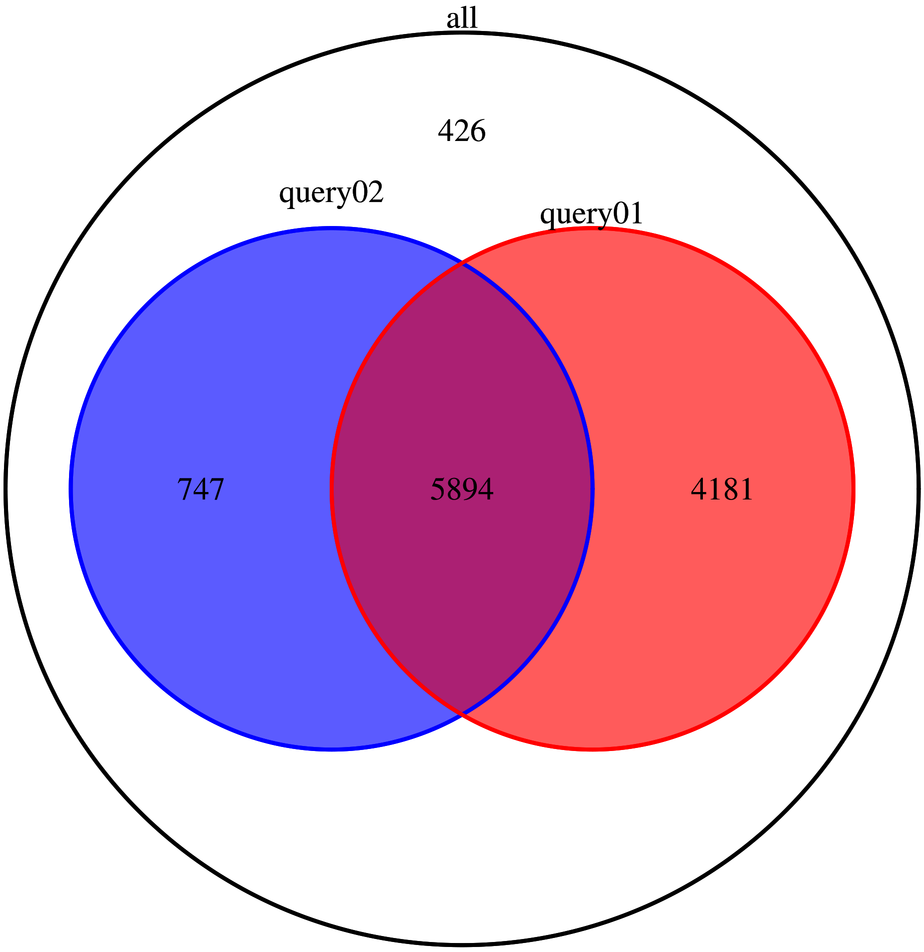

8: Comparison of all specified queries:

The following Chow Ruskey plot compares all queries
based on the besthits.
It represents an area–proportional Venn diagram,
revealing the distribution of peaks that could be annotated
per query and works for up to 5 queries.

Additional Info:
It is evalueated whether peaks are annotated,
but not whether they are annotated for the same feature

Chow Ruskey comparison of all peaks annotated with UROPA

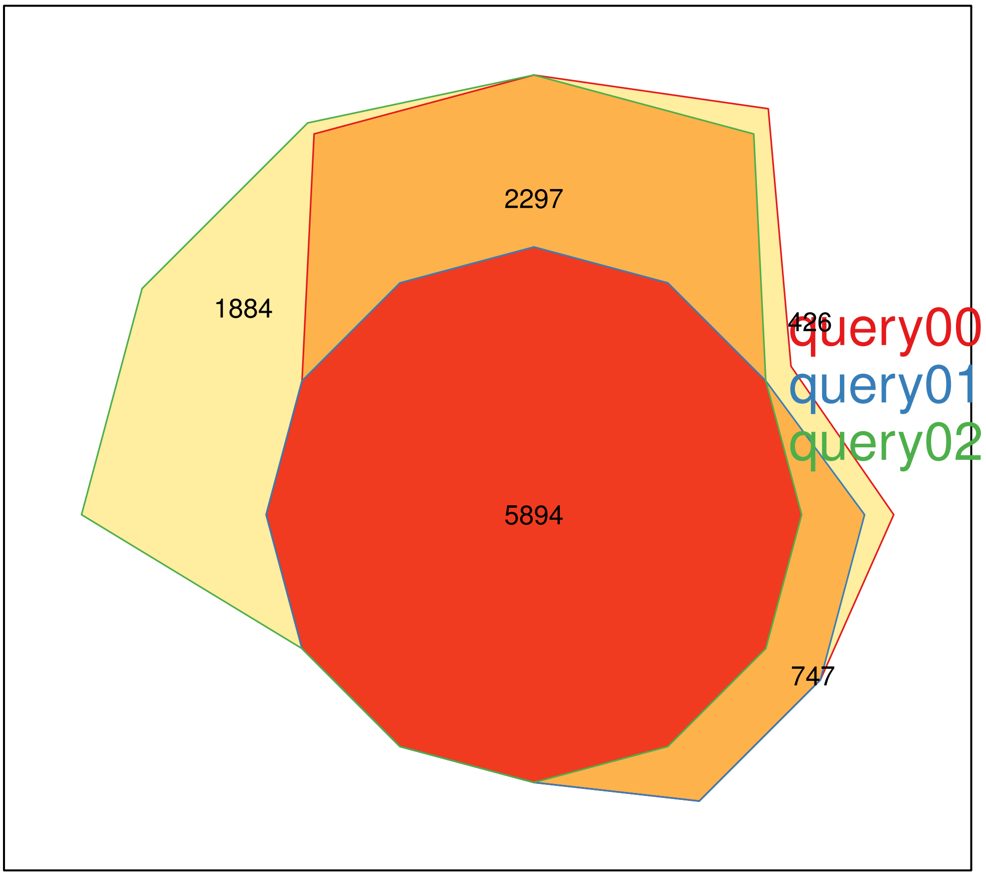

Additional File 2: Detailed comparison of UROPA and Goldmine with respect to distance.

Comparison of UROPA and Goldmine

UROPA Config:

{"queries":[{"feature":"gene","show.attributes":"gene_name", "internals":"T", "distance":100000}],

"gtf": " gencode.v19.annotation.gtf","bed": "ENCFF001VFA.bed"}

- Goldmine annotated 8 peaks more than UROPA (due to default distance, can be changed for UROPA)
- 7 peaks were annotated to different genes:

peak_id	chr	start	end	gm_location	gm_annotation	gm_distance	u_annotation	u_distance	u_location
peak_6230	chr15	62681868	62683020	intergenic	RP11-299H22.7	104662	TLN2	281	overlapStart
peak_10455	chr9	68726022	68726702	intergenic	CR786580.1	212834	RP11-391M20.1	267	overlapStart
peak_14776	chr5	111859512	111860357	intergenic	EPB41L4A-AS2	103201	RP11-159K7.1	22736	upstream
peak_14465	chr3	104240092	104242737	intergenic	AC016970.1	466451	RP11-281P11.1	19304	downstream
peak_3911	chr3	105974260	105977219	intergenic	Y_RNA	257524	RP11-93B21.1	68844	downstream
peak_14030	chrX	42801365	42803671	intergenic	RP3-326I13.1	232571	RP11-265D20.1	56905	upstream
peak_13485	chr2	62589854	62590700	intergenic	snoU13	97620	RP11-642D6.1	12403	upstream

- peak_6230 overlaps the start position of TLN2, but is annotated as intergenic with a distance of more than 100.000 by goldmine
- other annotated genes were not present in the online gencode version used by UROPA

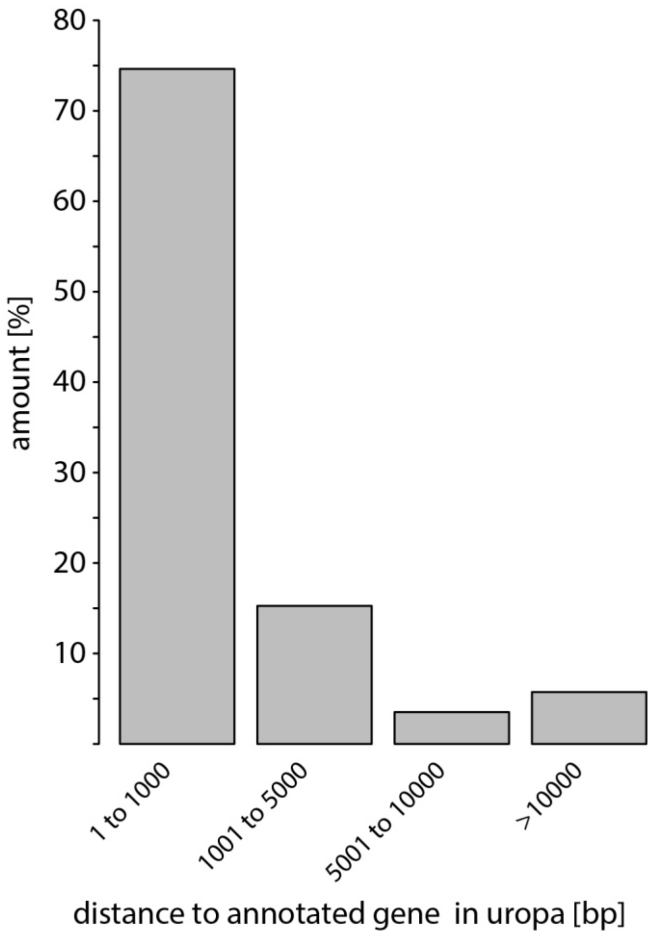

Comparison of peaks, called as promoters with a distance of 0 in goldmine, and respective distance in UROPA: In total, 10.559 peaks are called as promoters in goldmine, with same gene annotation in UROPA. Of these, UROPA reports

- 7881 with distance smaller 1000
- 1611 with distance 1000 to 5000
- 371 with distance 5000 to 10000
- 606 with distances above 10000 (more than 1% further than 50.000 bp off)

Examples:

- peak_11600 chr21 36419908 – 36421819
  - Goldmine: distance 0 in promoter of RUNX1
  - UROPA: peak inside feature, distance of 260.766 to feature
- peak_13322 chr8 99837692 - 99838615
  - Goldmine: distance 0 in promoter of STK3
  - UROPA: peak inside feature, distance of 116.902 to feature start

Additional File 3: Comparison of ChipPeakAnno and UROPA with *internals* parameter set to TRUE; activating the *additional* parameter for UROPA generates a smaller number of ChIPpeakAnno specific peaks, indicating that a large number of these are located outside the desired distance range.

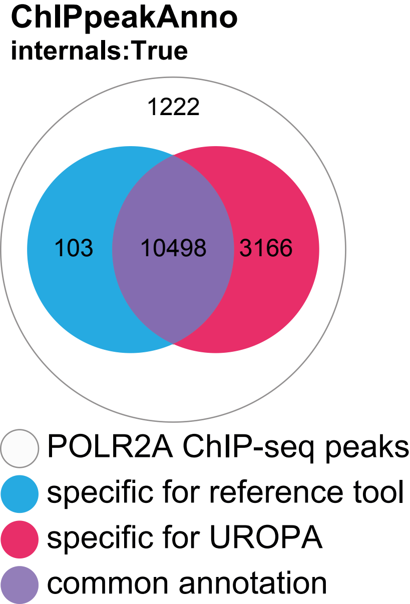
ChIPpeakAnno
internals:True
1222
103
10498
3166
POLR2A ChIP-seq peaks
specific for reference tool
specific for UROPA
common annotation

Additional File 4: Annotation statistics on a complex use case example deriving from colocalization of transcription factor binding sites (TFBS; peaks) with gene promoters (reference GTF) and ATAC-seq peaks (chromatin state open). The table represents the percentage of locations in cluster 1 (open chromatin) and 2 (closed chromatin) for four TFs respectively, defined by six different queries. Each query represents a different definition of a promotor region. Resulting numbers indicate that open locations (cluster 1) are located in close proximity to a gene (query 0-4), while closed locations (cluster 2) are depleted for proximity of coding genes.

query	feature - filter attribute	anchor	distance	Gabpa		Fli1		Klf1		Pax7	
				cluster 1	cluster 2	cluster 1	cluster 2	cluster 1	cluster 2	cluster 1	cluster 2
**0**	gene - protein coding	start	1000:500	85.5	4.21	88.11	3.41	84.42	2.52	30.71	1.06
**1**	gene - protein coding	start	2000:500	87.22	6.18	90.15	5.12	87.09	4.5	37.95	2.58
**2**	gene - protein coding	start	3000:500	88.2	7.87	90.83	6.72	88.21	6.39	40.68	4.09
**3**	gene - protein coding	start	5000:500	89.43	11.31	91.68	9.6	89.05	9.9	44.97	6.99
**4**	gene - pseudogene	start	5000:500	1.39	1.5	0.51	1.35	2.53	1.36	1.02	1.32
**5**	gene - protein coding	start	100000	99.51	77.6	100	73.25	99.86	77.73	95.66	73.14

all TF specific numbers in % of respective group, cluster 1 means open chromatin location, cluster 2 means closed chromatin location

Additional File 5: Runtime analysis with multiprocessing parameter; a large number of peaks (135k) and a complex configuration file was used multiple times with an increasing number of parallel threads (x axis) and runtime was recorded (y axis). The acceleration increases nearly linearly with the number of threads.

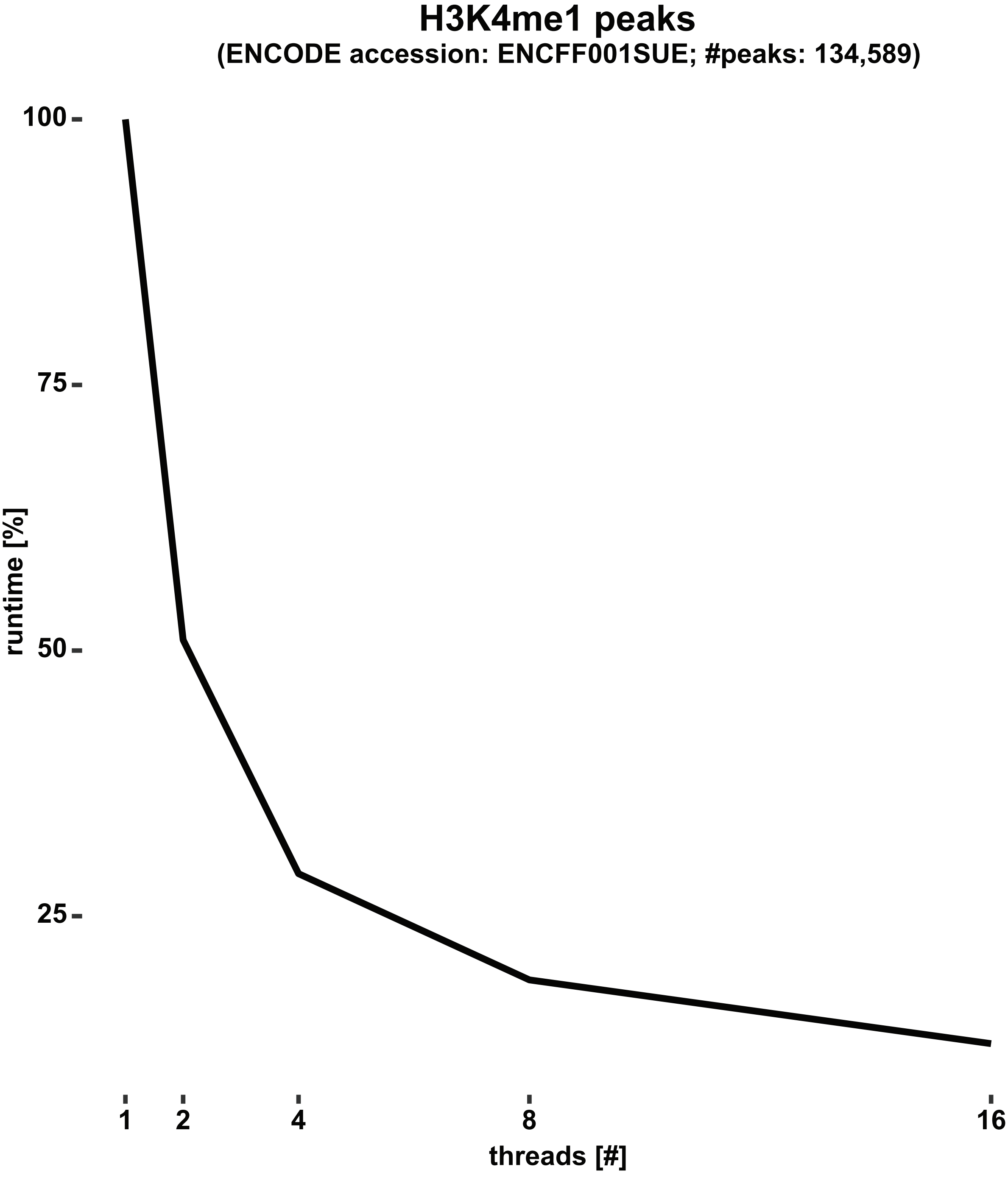


```
{
"queries":[
        {"feature":"gene", "distance":[5000,2000], "feature.anchor":"start", "filter.attribute":"gene_type",
                "attribute.value":"protein_coding","show.attributes":["gene_name","gene_type"]},
        {"feature":"gene", "distance":10000, "feature.anchor":"start", "direction":"upstream",
                "filter.attribute":"gene_type", "attribute.value":"protein_coding"},
        {"feature":"gene", "distance":1000, "internals":"True", "filter.attribute":"gene_type",
                "attribute.value":"protein_coding"},
        {"feature":"gene", "internals":"True", "filter.attribute":"gene_type",
                "attribute.value":"protein_coding"}
],
"priority": "T",
"gtf": "gencode.v19.annotation.gtf",
"bed": "ENCFF001SUE.bed"
}
```

Additional File 6: Influence of feature.anchor key with respect to gene assignment; UROPA was run twice with different configuration files for the same set of peaks deriving from histone modification H3K36me3. Resulting peak – gene annotations are visualized as a venn diagram. Approximately 25% of peaks were assigned to a different gene, driven by the feature.anchor effect.

H3K36me3: TSS vs. body

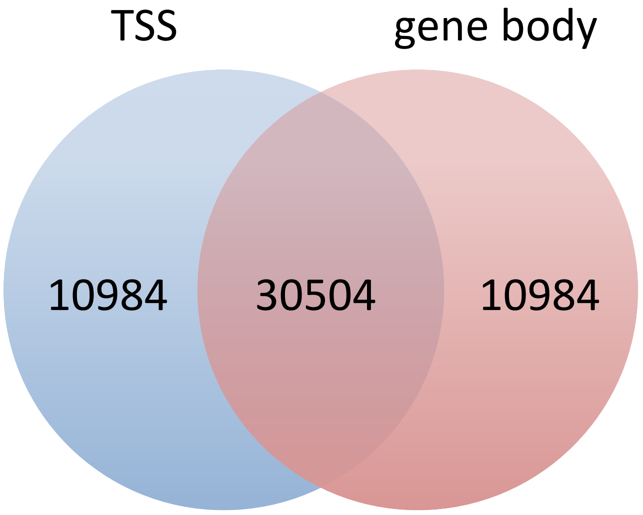

- GEO Sample GSM733733
- Human adult dermal fibroblasts
- H3K36me3
- 41488 peaks

- UROPA using either TSS or the gene body as anchor for annotation

- ~25% of peaks are annotated with a different gene

UROPA config: TSS

```
{
"queries":[
		{"feature":"gene","distance":100000, "show.attributes":["gene_id", "gene_name", "gene_type"], "feature.anchor" : ["start"]}
	],
"priority": "F",
"gtf": "/mnt/flatfiles/organisms/human/hg19_GRCh37/annotation/gencode/gencode.v25lift37.annotation_nochr.gtf",
"bed": "./GSM733733_hg19_wgEncodeBroadHistoneNhdfadH3k36me3StdPk.broadPeak_sorted.bed"
}
```

UROPA config: gene body

```
{
"queries":[
		{"feature":"gene","distance":100000, "show.attributes":["gene_id", "gene_name", "gene_type"], "feature.anchor" : [“center"]}
	],
"priority": "F",
"gtf": "/mnt/flatfiles/organisms/human/hg19_GRCh37/annotation/gencode/gencode.v25lift37.annotation_nochr.gtf",
"bed": "./GSM733733_hg19_wgEncodeBroadHistoneNhdfadH3k36me3StdPk.broadPeak_sorted.bed"
}
```
